# BIOGEN: evidence-grounded multi-agent reasoning framework for transcriptomic interpretation in antimicrobial resistance

**DOI:** 10.3389/fbinf.2026.1846404

**Published:** 2026-05-29

**Authors:** Elias Hossain, Mehrdad Shoeibi, Ivan Garibay, Niloofar Yousefi

**Affiliations:** Department of Industrial Engineering and Management Systems, University of Central Florida, Orlando, FL, United States

**Keywords:** antimicrobial resistance, biological reasoning, large language models, multi-agent framework, *Salmonella enterica*, transcriptomic interpretation

## Abstract

**Introduction:**

Interpreting gene clusters derived from RNA sequencing (RNA-seq) remains difficult in functional genomics, particularly in antimicrobial resistance studies where mechanistic context is needed for downstream hypothesis generation.

**Methods:**

We present BIOGEN, an evidence-grounded multi-agent framework for *post hoc* interpretation of RNA-seq transcriptional modules that integrates biomedical retrieval, structured interpretation, and multi-critic verification. BIOGEN organizes knowledge from PubMed and UniProt into traceable cluster-level explanations with explicit evidence reporting and confidence tiering.

**Results:**

On the primary *Salmonella enterica* dataset, BIOGEN achieved strong grounding and biological coherence, with BERTScore 0.689, Semantic Alignment Score 0.715, KEGG Functional Similarity 0.342, and a non-verifiable identifier rate of 0.000, compared with 0.100 for the LLM-only baseline. Across four additional bacterial RNA-seq datasets, BIOGEN preserved zero ungrounded outputs under the identifier-based criterion. In a controlled multi-dataset comparison against representative open-source agentic AI baselines, BIOGEN was the only framework that consistently produced zero non-verifiable identifier outputs across all five datasets.

**Discussion:**

These results indicate that retrieval access alone is insufficient to ensure reliable biological interpretation. Evidence-grounded orchestration is essential for transparent, source-traceable transcriptomic reasoning under distribution shift.

## 1 Introduction

Interpreting RNA-seq gene clusters remains a central challenge in transcriptomics (Kiselev et al., 2019; Williams et al., 2014; Finotello and Di Camillo, 2015), with continuing methodological development in single-cell and bulk RNA-seq cluster interpretation in recent years (Vandereyken et al., 2023; Zhang et al., 2023). Clustering methods such as K-means and spectral clustering can group genes with similar expression patterns into transcriptional modules, but the downstream biological interpretation of these modules still relies primarily on enrichment-based analyses, including Gene Ontology over-representation analysis and Gene Set Enrichment Analysis (GSEA) (Subramanian et al., 2005; Zhao and Rhee, 2023; Chen et al., 2023). These methods provide useful summaries at the level of predefined pathways or categories, yet they often offer limited insight when signals are weak, diffuse, or poorly represented in existing annotation resources (Subramanian et al., 2005; Zhao and Rhee, 2023). As a result, cluster-specific mechanistic interpretation frequently still depends on manual literature review and expert curation, which limits scalability, reproducibility, and traceable hypothesis generation (Subramanian et al., 2005; Chen et al., 2023).

Large language models (LLMs) have recently emerged as powerful tools for biomedical text synthesis and knowledge-intensive reasoning (Singhal et al., 2023; Tian et al., 2024). However, fluency alone does not ensure factual reliability. When used without external grounding, LLMs can generate plausible but unsupported biological statements, omit verifiable provenance, and introduce hallucinated content, including fabricated or misleading references (Roustan and Bastardot, 2025). Retrieval-augmented generation (RAG) addresses part of this limitation by conditioning generation on external evidence rather than relying solely on parametric memory (Lewis et al., 2020). Recent biomedical reviews similarly suggest that retrieval-augmented workflows can improve factuality and updateability, while also emphasizing continuing challenges in evaluation, attribution, and reliability (Liu et al., 2025). Even so, evidence-grounded, role-specialized agentic systems for transcriptomic cluster interpretation remain underexplored, especially in settings that require explicit critic-based verification and direct comparison with conventional enrichment pipelines.

This gap is particularly important in antimicrobial resistance research involving *Salmonella enterica*, a major foodborne pathogen associated with a growing global resistance burden and substantial implications for public health, food safety, and treatment effectiveness (Kumar et al., 2025; Wang et al., 2025). RNA-seq can reveal genome-wide transcriptional programs related to virulence, resistance, and metabolic adaptation, but interpreting these programs still depends largely on statistical summaries followed by manual biological contextualization (Chen et al., 2023; Stévenin et al., 2024). A framework that systematically links gene clusters to verifiable evidence, while also quantifying what is recovered beyond standard enrichment analysis, could therefore support more reliable and reproducible biological hypothesis generation.

To address this need, we introduce BIOGEN, a role-specialized multi-agent framework for evidence-grounded interpretation of RNA-seq transcriptional modules. BIOGEN formulates interpretation as a verification-constrained reasoning process involving three specialist components: (i) a Retriever that gathers supporting evidence from PubMed and UniProt, (ii) an Interpreter that produces cluster-level explanations grounded in retrieved sources, and (iii) a set of Critic Agents that evaluate factual grounding, semantic coherence, and adversarial consistency. BIOGEN also employs a data-adaptive evidence-tiering strategy that assigns interpretations to High confidence, Moderate confidence, or Suggestive categories based on the empirical distribution of consensus critic scores, thereby avoiding manually selected thresholds.

BIOGEN is intended as an interpretive support layer rather than a standalone discovery engine. It does not claim novel biological discoveries or replace experimental validation. Instead, it organizes existing biomedical knowledge into transparent, cluster-specific interpretations that complement standard transcriptomic workflows and make the evidential basis of each interpretation explicit. Although BIOGEN is methodologically applicable to broader bacterial RNA-seq interpretation, the present evaluation is scoped to antimicrobial-resistance-relevant bacterial transcriptomic datasets, namely, the primary multi-omics AMR cohort in *S. enterica* and four external bacterial-stress datasets that involve antibiotic perturbation, biofilm formation, and stress-response programs.

The main contributions of this work are as follows:

1. We formulate *post hoc* RNA-seq cluster interpretation as an evidence-grounded, verification-aware reasoning problem and introduce BIOGEN, a modular multi-agent framework that integrates retrieval, structured interpretation, and critic-based validation.
2. We present a reproducible local implementation that combines per-cluster gene selection, literature and protein retrieval, data-adaptive evidence tiering, and traceable cluster-level reporting without relying on proprietary inference services.
3. We evaluate BIOGEN across five bacterial RNA-seq datasets using grounding- and relevance-oriented metrics, including BERTScore, Semantic Alignment Score, KEGG Functional Similarity where available, the identifier-based hallucination rate (defined as the proportion of interpretations lacking a verifiable PMID, DOI, or UniProt accession; Section 5.3.4), and evidence-tier distributions, and analyze how retrieval and critic modules affect identifier-level source traceability and interpretive reliability under distribution shift.
4. We compare BIOGEN with standard enrichment baselines and representative open-source agentic AI frameworks under a shared local setup, showing that BIOGEN consistently provides the strongest identifier-level traceability and produces zero non-verifiable identifier outputs across datasets under the strict identifier-based criterion, while generic agentic frameworks may achieve higher similarity-based scores but with substantially weaker grounding reliability.

The remainder of the paper is organized as follows. Section 2 reviews related work. Sections 3, 4 introduce the BIOGEN framework and its design rationale. Section 5 describes the experimental setup. Section 6 presents results on the primary dataset, design sensitivity analyses, complementarity with enrichment analysis, cross-organism generalization, and comparison with representative open-source agentic AI frameworks. Finally, Sections 7, 8 discuss limitations, implications, and future directions.

## 2 Related work

Interpretation of RNA-seq gene expression data remains a longstanding challenge in transcriptomics, including in antimicrobial resistance research. Conventional transcriptomic analysis typically progresses from differential expression or clustering to pathway- or ontology-based enrichment methods such as over-representation analysis and Gene Set Enrichment Analysis (GSEA) (Subramanian et al., 2005). Although these methods are statistically well grounded and widely used, they summarize results at the level of predefined gene sets and may offer limited biological insight when signals are weak, diffuse, or incompletely represented in annotation resources. In addition, gene-level analysis may yield either no individually significant genes or long lists of genes without a clear unifying theme, leaving interpretation dependent on substantial expert curation.

In parallel, machine learning methods have been applied extensively to antimicrobial resistance prediction and related genomic tasks. Sequence-based language models, feature-based predictive models, and comparative genomics pipelines have contributed to resistance detection, susceptibility prediction, and evolutionary analysis (Zhang et al., 2024; You et al., 2025; Ayoola et al., 2024; Wheeler et al., 2018). However, these approaches are primarily designed for prediction or classification rather than for *post hoc* interpretation of transcriptional modules grounded in curated evidence.

More recently, large language models have been explored for biomedical annotation, clinical reasoning, and antimicrobial resistance interpretation (Giske et al., 2024). Domain-specific biomedical agent systems have likewise been proposed for tasks such as clinical decision support and multi-omics reasoning. However, some of these systems rely on proprietary models or partially closed pipelines, which complicates direct reproducibility and fair benchmarking. More importantly, LLM-based biomedical systems remain vulnerable to hallucination, incomplete source attribution, and limited traceability when explicit validation mechanisms are absent.

A central limitation in the current literature is that LLM-based interpretation is rarely evaluated directly against standard enrichment workflows. Existing studies often either replace enrichment analysis with free-form generation or use LLMs as annotation tools without quantifying what additional biological coverage they provide beyond pathway-based baselines. In this work, we address that limitation by treating the two approaches as complementary and explicitly measuring the additional cluster-level biological coverage that evidence-grounded agentic interpretation can provide relative to KEGG over-representation analysis.

Structured gene-signature resources such as MSigDB (Liberzon et al., 2011) further highlight the continuing importance of curated knowledge bases in transcriptomic interpretation. Although BIOGEN currently retrieves evidence from PubMed and UniProt, integrating structured gene-set resources such as MSigDB represents a natural extension that could improve evidence specificity and broaden the range of recoverable cluster-level associations.

Overall, BIOGEN is positioned as an interpretive layer rather than a replacement for standard transcriptomic analysis. The framework combines structured retrieval, local LLM-based synthesis, and multi-critic verification to produce transparent, evidence-traceable explanations for RNA-seq gene clusters that complement existing enrichment workflows. In addition, the present study emphasizes reproducible comparison against open-source baseline systems under a shared local setup, which is especially important in a landscape where some biomedical agent frameworks rely partly on proprietary or otherwise non-reproducible infrastructure.

## 3 Preliminaries and motivation

BIOGEN is designed to support reliable post hoc interpretation of RNA-seq gene clusters by organizing and validating existing biomedical evidence. The framework operates on cluster outputs produced by standard transcriptomic workflows and is intended specifically for downstream biological interpretation, rather than for the development of new predictive or clustering methods.

Let $M\in R^{s\times g}$ denote a normalized RNA-seq expression matrix with s samples and $g$ genes. Instead of clustering samples, BIOGEN operates on the transposed matrix $M^{⊤}\in R^{g\times s}$ , where each row represents the expression profile of a gene across samples. A clustering procedure partitions the gene set into k disjoint transcriptional modules $G=\left\{G_{1},\ldots ,G_{k}\right\}$ , where each $G_{i}\subseteq \left\{1,\ldots ,g\right\}$ denotes a set of co-expressed gene identifiers. These modules serve as the primary units of interpretation.

For each cluster $G_{i}$ , representative genes are selected according to within-cluster variance rather than global variance. Specifically, the top- v genes are those with the highest variance across samples within that cluster, so that downstream interpretation is anchored in signals that are locally informative for the cluster under consideration.

The overall interpretive process can be written as

$$F\left(G_{i}\right)=f_{C}\;\left(f_{I}\;\left(f_{R}\left(G_{i}\right)\right)\right),$$

where $f_{R}$ , $f_{I}$ , and $f_{C}$ denote the Retriever, Interpreter, and Critic components, respectively. Intermediate outputs are stored in a shared cache to improve traceability and reduce redundant retrieval.

Given a cluster $G_{i}$ , the Retriever $f_{R}$ queries external biomedical resources and returns an evidence set

$$E_{i}=f_{R}\left(G_{i}\right),$$

which consists of literature abstracts and protein annotations from PubMed and UniProt, each associated with explicit identifiers when available. The Interpreter $f_{I}$ then synthesizes this evidence into a cluster-level interpretation

$$T_{i}=f_{I}\left(E_{i}\right),$$

which summarizes putative biological themes while remaining constrained to the retrieved source material.

The Critic ensemble $f_{C}$ evaluates $T_{i}$ with respect to factual grounding, semantic consistency, and adversarial robustness, producing an aggregated confidence score ${\bar{s}}_{i}\in \left[0,1\right]$ . Rather than imposing a manually selected global threshold, BIOGEN derives evidence-tier boundaries from the empirical distribution of ${\left\{{\bar{s}}_{i}\right\}}_{i=1}^{k}$ using the 33rd and 66th percentiles. Interpretations are then labeled High confidence, Moderate confidence, or Suggestive according to their relative position within that distribution.

## 4 BIOGEN framework

BIOGEN is a modular multi-agent framework for post hoc interpretation of RNA-seq transcriptional modules. It operates on gene clusters produced by an upstream transcriptomic workflow and transforms them into structured, evidence-grounded biological explanations. The framework is built around three guiding principles: systematic use of external biomedical evidence, transparent intermediate reasoning, and critic-based verification before final reporting.

**FIGURE 1:**
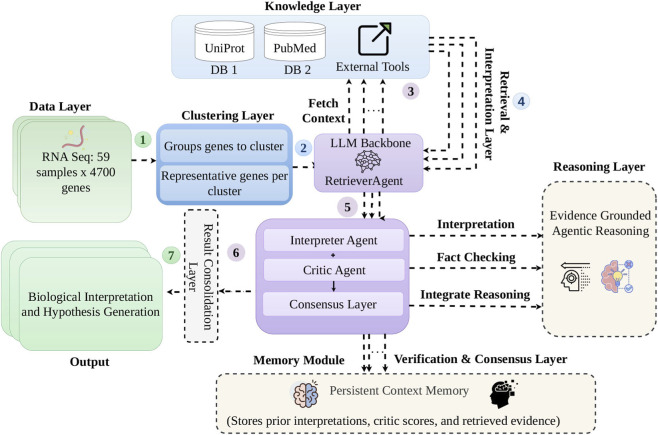
Overview of the BIOGEN framework for interpretable RNA-seq analysis. Gene-level clusters are enriched with PubMed and UniProt evidence, interpreted by the Interpreter Agent using a local open-source LLM, and evaluated through a panel of Critic Agents, producing transparent, literature-grounded outputs with data-adaptive evidence-tier labels.

At a high level, BIOGEN takes a gene cluster as input, retrieves relevant supporting evidence from biomedical resources, synthesizes that evidence into a structured cluster-level interpretation, and then evaluates the resulting explanation through multiple critics before assigning a confidence score and evidence tier. Figure 1 summarizes this workflow and illustrates the interaction among the clustering input, retrieval and interpretation modules, critic-based validation, and the final literature-grounded output.

### 4.1 Core components

BIOGEN consists of six functional components: an input layer, a retrieval layer, an interpretation layer, a verification layer, an evidence-tier assignment module, and an output layer. A lightweight memory module supports traceability and reuse of intermediate results throughout the pipeline. For presentation clarity, these components are grouped into two broader stages: input and evidence acquisition, followed by interpretation, verification, and output generation.

#### 4.1.1 Input and evidence acquisition

The framework accepts a set of gene clusters $\left\{G_{1},\ldots ,G_{m}\right\}$ derived from gene-level clustering of the expression matrix. No assumptions are made about the specific upstream clustering method. This design decouples interpretive reasoning from statistical partitioning and allows BIOGEN to remain compatible with a range of transcriptomic workflows. Each cluster is treated as a fixed unit of downstream interpretation.

For each cluster $G_{i}$ , the Retriever Agent issues structured queries to PubMed and UniProt using representative gene identifiers from that cluster. The purpose of this step is to assemble an evidence pool containing literature abstracts, protein annotations, and associated database identifiers. Retrieved materials are stored in structured form and cached for reuse. The retrieval layer functions strictly as an evidence collection module and does not itself generate biological claims. The contribution of retrieval-source selection under this design is examined empirically in the design-sensitivity analyses presented in Section 6.2.

#### 4.1.2 Interpretation, verification, and output

The *Interpreter Agent* synthesizes the retrieved evidence into a cluster-level textual interpretation. The output is structured to summarize functional themes, putative pathways, regulatory context, and explicit limitations. Interpretations are constrained to the retrieved sources and are not intended to introduce unsupported biological claims. A local open-source LLM, Mistral-7B-Instruct-v0.2 (Jiang et al., 2023) with 4-bit NF4 quantization, serves as the generation backbone in order to reduce dependence on external services and improve reproducibility.

Candidate interpretations are then evaluated by three *Critic Agents*, each of which examines the output from a different perspective. The *Evidence-Strict Critic* performs rule-based factual validation against retrieved identifiers, the *Semantic Critic* uses embedding-based similarity to assess alignment with the evidence pool, and the *Adversarial Critic* applies counterfactual probing to expose potentially unsupported claims. These critics do not rewrite the interpretation. Instead, they produce scores and reliability judgments that are later aggregated into a consensus assessment.

After all clusters have been evaluated, consensus confidence scores are pooled and converted into evidence tiers using data-adaptive thresholds. Specifically, the 33rd and 66th percentiles of the score distribution define the boundaries between *Suggestive*, *Moderate confidence*, and *High confidence* outputs. This percentile-based strategy avoids reliance on a fixed threshold that may not transfer well across organisms or experimental conditions. The empirical stability of this percentile-based tiering scheme, together with additional analyses of retrieval-source behavior and evidence specificity, is examined later in Section 6.2.

A structured cache stores retrieved evidence, intermediate interpretations, and critic evaluations. This memory module supports traceability, reduces redundant retrieval, and preserves intermediate results for later inspection. The final output for each cluster consists of a structured biological interpretation, explicit PubMed and UniProt references when available, a consensus confidence score, and an evidence-tier label. These outputs are intended to support biological contextualization and hypothesis prioritization rather than replace experimental validation.

### 4.2 Design rationale

The ordering of BIOGEN’s components was chosen to preserve traceability between retrieved evidence and the final interpretation. Retrieval is performed before generation so that the Interpreter Agent operates on an explicit evidence pool rather than relying on parametric memory alone. This design reduces dependence on unsupported internal associations and ensures that candidate interpretations can be linked back to identifiable external sources.

The critic ensemble is applied after interpretation rather than before generation because the critics are intended to evaluate the complete cluster-level explanation, including its factual grounding, semantic consistency, and reporting quality. In this role, the critics assess not only whether evidence has been retrieved, but also whether that evidence has been synthesized into a coherent and sufficiently supported biological interpretation. Applying critics only at the retrieval stage would not capture this explanation-level behavior. At the same time, the current design does not preclude alternative orderings. Earlier verification of retrieved evidence, integration of structured signature databases such as MSigDB, or iterative critic-in-the-loop generation may all be useful extensions for future work. The empirical implications of these design choices are examined later in Section 6.2.

### 4.3 Reliability aggregation

For a given gene cluster, multiple Critic Agents independently evaluate factual correctness, semantic consistency, and adversarial robustness. Let $s_{ij}$ and $r_{ij}$ denote the confidence score and binary reliability assignment produced by critic j for interpretation i , where j∈{1,…,m} indexes the critics in the ensemble (with m=3 in the present implementation). The aggregated confidence score is defined as

$${\bar{s}}_{i}=\frac{1}{m}\overset{m}{\underset{j=1}{\sum }}s_{ij},$$

and the final reliability indicator ${\bar{r}}_{i}$ is determined by majority voting over the per-critic binary assignments $r_{ij}$ :

$${\bar{r}}_{i}=I\;\left[\overset{m}{\underset{j=1}{\sum }}r_{ij}>\frac{m}{2}\right],$$

where each $r_{ij}\in \left\{0,1\right\}$ is the binary reliability flag emitted by critic j (so the phrase “critic-level binary assignment” refers to this per-critic Boolean output, and “majority voting” means that the majority of the m critics must return $r_{ij}=1$ ). Concretely, each critic j emits a continuous score $s_{ij}\in \left[0,1\right]$ and a binary reliability flag $r_{ij}=I\left[s_{ij}\geq \theta_{j}\right]$ , where $\theta_{j}$ is a per-critic operating threshold set to $\theta_{\text{evidence}}=0.7$ , $\theta_{\text{semantic}}=0.6$ , and $\theta_{\text{adversarial}}=0.5$ . These thresholds are operating points rather than calibrated probabilities: each $s_{ij}$ should be interpreted as an internal reliability score produced by a single critic under its own scoring rubric (rule-based identifier matching for the Evidence-Strict critic; LLM-judged JSON outputs for the Semantic and Adversarial critics), not as a calibrated estimate of the probability that the interpretation is correct. The thresholds were chosen so that the Evidence-Strict critic, which counts gene-specific PubMed hits, requires the majority of cluster genes to be supported by a specific PubMed hit before it returns a positive flag, while the LLM-judged critics use lower per-critic thresholds to avoid degenerate all-true or all-false votes when the LLM judge is uncertain.

The aggregated score ${\bar{s}}_{i}$ is the unweighted mean of the per-critic scores. Aggregation by mean rather than by minimum was chosen because the three critics evaluate complementary aspects of the same interpretation–a single critic flagging a weakness should reduce confidence but should not unilaterally veto agreement from the other two. The reliability flag ${\bar{r}}_{i}$ is determined by majority vote over the binary $r_{ij}$ values; with three critics, ${\bar{r}}_{i}=1$ iff at least two of the three per-critic flags are positive. This decouples the continuous reliability signal ${\bar{s}}_{i}$ used for evidence tiering from the binary reliability indicator ${\bar{r}}_{i}$ used for cluster-level reporting, so that a cluster can be flagged as “unreliable by majority vote” but still receive a meaningful within-run tier label.

Evidence tiers are then assigned according to the score distribution across the full run:

$$Tier\left(i\right)=\left\{\begin{array}{cc} Highcon\mathrm{fi}dence & \text{if }{\bar{s}}_{i}\geq p_{66},\\ Moderatecon\mathrm{fi}dence & \text{if }p_{33}\leq {\bar{s}}_{i}<p_{66},\\ Suggestive & \text{if }{\bar{s}}_{i}<p_{33}, \end{array}\right.$$

where $p_{33}$ and $p_{66}$ denote the 33rd and 66th percentiles of ${\left\{{\bar{s}}_{i}\right\}}_{i=1}^{k}$ , and k is the number of gene clusters produced by the upstream clustering procedure (in this work, k=10 ; see Section 5.2). Because the percentiles are derived from the empirical distribution of consensus scores within each pipeline run, evidence-tier labels are dataset-relative rankings rather than absolute confidence levels. A “High confidence” label on one dataset is not directly comparable to a “High confidence” label on another dataset, and we emphasize this caveat in every cross-dataset table.

### 4.4 Algorithm

Algorithm 1 summarizes the end-to-end workflow of BIOGEN. The pipeline begins by initializing the cache, runtime environment, and agent instances. Gene-level clustering is then performed on the transposed expression matrix, and representative genes for each cluster are selected according to within-cluster variance. Evidence is retrieved for each representative gene, with cached results reused whenever available to reduce redundant queries. The Interpreter Agent then produces a structured cluster-level interpretation $T_{i}$ together with two placeholder values $\left(c_{i},r_{i}\right)$ : $c_{i}$ is the LLM’s self-reported confidence score for interpretation i (a value in [0,1] that the LLM may or may not produce, treated as a placeholder), and $r_{i}$ is a corresponding placeholder reliability flag. Both placeholders are subsequently overwritten by the consensus aggregates ${\bar{s}}_{i}$ and ${\bar{r}}_{i}$ produced by the Critic ensemble (Section 4.3); they appear in line 13 of Algorithm 1 only to keep the LLM-output triple compatible with the Interpreter Agent’s interface and are not used directly for tier assignment or final reporting. After all clusters have been processed, evidence-tier thresholds are derived from the full score distribution, and the final labeled outputs are saved.

Algorithm 1 BIOGEN: Complete Agentic Pipeline.

Require: RNA-seq matrix M , number of clusters k , max evidence per gene r

Ensure: Cluster interpretations I

1: Initialize cache C , runtime environment, and retrieval clients
2: Instantiate cluster, retriever, interpreter, and critic agents
3: $\left(M^{\prime },y\right)\leftarrow A_{\text{cluster}}.run\left(M^{⊤}\right)$ ⊳ gene-level clustering
4: S←{} ⊳ collect scores for tier derivation
5: **for** i←1 to k **do**
6: $G_{i}\leftarrow$ top- v genes by within-cluster variance
7:  **for all** $g\in G_{i}$ **do**
8:   **if** g∈C **then** retrieve from cache
9:   **else** Retrieve and cache evidence (up to r items)
10:   **end** **if**
11:  **end** **for**
12:  Construct structured prompt $P_{i}$

13: $\left(T_{i},c_{i},r_{i}\right)\leftarrow A_{\text{interp}}.generate\left(P_{i}\right)$

14:  **for all** critic j in critic ensemble **do**
15:   Evaluate $T_{i}$ ; store $\left(s_{ij},r_{ij}\right)$

16:  **end for**
17: ${\bar{s}}_{i}\leftarrow \frac{1}{m}\sum_{j}s_{ij}$ ; ${\bar{r}}_{i}\leftarrow \text{majority}\left(\left\{r_{ij}\right\}\right)$

18: $S\leftarrow S\cup \left\{{\bar{s}}_{i}\right\}$

19: **end for**
20: $p_{33},p_{66}\leftarrow \text{percentile}\left(S,33\right),\;\text{percentile}\left(S,66\right)$

21: **for** i←1 to k **do**
22:  Assign evidence tier from ${\bar{s}}_{i}$ , $p_{33}$ , and $p_{66}$

23:  Post-process $T_{i}$ and save to I

24: **end for**
25: **return** I

## 5 Experimental setup

The experimental evaluation was designed to assess five aspects of BIOGEN: evidence grounding on the primary Salmonella enterica dataset, sensitivity to key architectural design choices, complementarity with conventional enrichment analysis, generalization across additional bacterial RNA-seq datasets, and behavior relative to representative open-source agentic AI frameworks. The overall workflow of the framework is described in Section 4 and summarized in Algorithm 1. This section presents the data sources and preprocessing procedures (Sections 5.1, 5.2), the evaluation metrics (Section 5.3), and the controlled settings used in the agentic baseline comparison (Section 5.4). Additional implementation details are provided in the appendix. Together, these sections define the shared experimental conditions underlying the primary-dataset evaluation, design-sensitivity analyses, cross-organism analysis, and agentic framework comparison.

### 5.1 Data sources

The primary RNA-seq dataset used in this study was obtained from the European Nucleotide Archive under accession PRJEB67574 and originates from the multi-omics investigation of Stévenin et al. (Stévenin et al., 2024). It contains transcriptomic profiles from 58 *Salmonella enterica* isolates and provides a biologically well-characterized setting for evaluating cluster-level interpretation.

To assess the robustness and generalizability of the framework beyond the primary cohort, we additionally incorporated four publicly available bacterial RNA-seq datasets from the Gene Expression Omnibus (GEO). These external datasets were selected according to four criteria: relevance to antimicrobial resistance or bacterial stress-response biology, public availability of gene-level count data, sufficient sample size for cluster-based analysis, and compatibility of gene identifiers with downstream functional annotation resources.

The first external dataset, GSE251671, is a large-scale transcriptional compendium for *P. aeruginosa* UCBPP-PA14 generated under antibiotic perturbation and CRISPRi target depletion (Romano et al., 2024). It contains 1,059 samples and provides processed gene-level count data suitable for direct downstream analysis. The second dataset, GSE144604, was generated from *E. coli* MG1655 and profiles transcriptional responses to combinations of antibiotics and biocides, thereby capturing patterns of cross-protection and cross-vulnerability relevant to antimicrobial stress (Wang et al., 2020). This dataset contains 135 samples. The third dataset, GSE224463, is an *Escherichia coli* K-12 time-course study examining wild-type and ΔrpoS responses across multiple stress conditions, including stationary-phase transition, osmotic stress, and low temperature (Adams et al., 2023). It contains 192 samples. The fourth dataset, GSE55197, is a *Pseudomonas aeruginosa* PA14 transcriptomic compendium spanning 14 environmental conditions, including biofilm growth, anaerobic growth, osmotic variation, and nutrient limitation (Dötsch et al., 2015), which contains 47 samples.

Together, these datasets introduce variation in organism, experimental design, and transcriptomic context while remaining relevant to the broader problem of bacterial functional interpretation.

### 5.2 Preprocessing

For the primary *Salmonella* dataset, raw sequencing reads were processed using a standard RNA-seq workflow. FastQC was used for initial quality assessment, fastp for adapter trimming and filtering, BWA-MEM for alignment to the *S. enterica* Typhimurium LT2 reference genome (RefSeq GCF_000006945.2), samtools for alignment processing, and featureCounts for gene-level quantification. This procedure produced a final expression matrix containing 58 samples and 4,679 genes.

In contrast, the four external validation datasets were obtained in processed count form and therefore did not require re-alignment from raw FASTQ reads. For each dataset, the released count tables were converted into a standardized matrix representation with genes as rows, samples as columns, and a unified gene_id field. When the deposited processed data consisted of one count table per sample, these files were merged by shared gene identifier after removal of non-biological summary rows. Only datasets satisfying the minimum criteria of at least 20 samples and approximately 2,000 or more genes in the final matrix were retained for downstream validation. Full download and harmonization scripts are provided in the project repository.

To identify transcriptionally coherent modules, clustering was performed on the transposed expression matrix so that genes, rather than samples, served as the primary clustering units. This design aligns with the biological objective of identifying co-expressed genes that may share regulatory or functional relationships. K-means clustering with k=10 was applied, with the number of clusters selected using the elbow criterion based on within-cluster inertia. Representative genes for each cluster were selected according to within-cluster variance rather than global variance, thereby ensuring that each cluster interpretation was driven by its most informative local signals. The resulting clusters were treated as fixed inputs to BIOGEN throughout all experiments.

We acknowledge that fixing k=10 across five datasets that differ in organism, sample size, and number of genes is a deliberate simplification rather than a per-dataset optimum. The choice was motivated by three considerations. First, BIOGEN is designed as an interpretive layer that operates on externally supplied gene clusters; the cluster count is therefore a property of the upstream clustering step rather than of the framework itself, and holding k fixed isolates the contribution of the interpretive layer across datasets. Second, on each of the five datasets, the within-cluster inertia profile was approximately monotone-decreasing without a sharp single elbow, and k∈[8,12] produced visually similar inertia values; k=10 falls inside this range for every dataset and is therefore a defensible fixed setting for cross-dataset comparison. Third, a per-dataset k would introduce an additional source of variability when comparing identifier-level grounding behavior across datasets, which would weaken the cross-dataset comparison rather than strengthen it. We note that organism-specific clustering–including non-K-means methods, stability-aware k selection, and graph-based clustering–is a natural extension and is flagged in Section 7 as future work.

### 5.3 Evaluation metrics

Evaluation was conducted using complementary metrics drawn from the natural language processing and biomedical literature. These measures assess semantic correspondence, biological relevance, factual traceability, and interpretive reliability from different perspectives. In the final unified multi-dataset analysis, we emphasize metrics that can be computed consistently across datasets and framework settings, namely, BERTScore, Semantic Alignment Score (SAS), KEGG Functional Similarity where available, the identifier-based hallucination rate (Section 5.3.4; also referred to as the non-verifiable identifier rate), and evidence-tier distributions.

#### 5.3.1 BERTScore

BERTScore (Zhang et al., 2019) was used to quantify the semantic overlap between generated interpretations and retrieved evidence through contextual token embeddings. We report the F1 variant computed with the distilbert-base-uncased backbone:

$$BERTScore=F_{1}\left(v_{\text{interpretation}},\;v_{\text{evidence}}\right),$$

where v denotes token-level contextual embeddings. Higher values indicate stronger semantic overlap between the generated interpretation and the available evidence context. We use BERTScore rather than word-overlap metrics such as BLEU, ROUGE, or METEOR because Zhang et al. (Zhang et al., 2019) report that contextual-embedding similarity correlates more strongly with human judgments than n -gram overlap on a range of generation tasks. In our setting, where retrieved evidence and cluster interpretations frequently share semantic content but use different surface lexicalizations (for example, “efflux pump” *versus* “multidrug-resistance transporter”), n -gram-overlap metrics would penalize valid paraphrasing more aggressively than BERTScore.

#### 5.3.2 Semantic Alignment Score

The Semantic Alignment Score (SAS) measures cosine similarity between Sentence-BERT embeddings of the generated interpretation and the retrieved evidence pool:

$$SAS=\cos\left(v_{\text{evidence}},\;v_{\text{interpretation}}\right),$$

where embeddings are computed using the all-MiniLM-L6-v2 Sentence-BERT model (Reimers and Gurevych, 2019). This metric reflects overall semantic proximity between the interpretation and the evidence. Because evidence density and specificity vary across organisms and datasets, SAS is most informative as a relative alignment signal within a given dataset and should be interpreted cautiously in cross-dataset settings.

#### 5.3.3 KEGG Functional Similarity

To assess biological relevance, we compute the semantic similarity between each cluster interpretation and the KEGG functional descriptions associated with the representative genes in that cluster. Gene-function descriptions are retrieved through the KEGG REST API using organism-specific KEGG codes where available, and similarity is computed with Sentence-BERT embeddings. This metric serves as an external proxy for biological plausibility by comparing generated interpretations against curated functional annotations. Because KEGG annotation coverage varies across organisms and datasets, this metric is reported only where reliable mapping is available.

#### 5.3.4 Identifier-based source traceability (non-verifiable identifier rate)

We measure hallucination operationally as the absence of identifier-based source traceability in the generated interpretation. The metric is defined as

$$H=1-\frac{\left|R_{\text{verified}}\right|}{\left|R_{\text{total}}\right|},$$

where $R_{\text{verified}}$ denotes interpretations containing at least one verifiable identifier in explicit citation form, such as a PMID, DOI, or UniProt accession, and $R_{\text{total}}$ denotes the total number of interpretations. Because each cluster-level interpretation is a multi-sentence document (typically 5–15 sentences spanning the structured sections *Functional Themes*, *Putative Pathways*, *Key Genes and Evidence*, *References*, and *Limitations*), the metric operates at the document level: an interpretation is counted as verified iff at least one regex-formatted identifier match is present anywhere in the entire interpretation text. We deliberately chose this document-level definition rather than a per-sentence definition because (i) the structured prompt asks the LLM to enumerate references in a single section near the end of the interpretation, so per-sentence counting would systematically under-credit interpretations whose claims are supported by a centrally listed reference block, and (ii) per-sentence verification would require sentence-level claim alignment, which is itself an open research problem that goes beyond identifier-level traceability. The cost of the document-level definition is that an interpretation containing one valid identifier and several unverified sentences would still be counted as verified; we therefore present this metric as an identifier-level lower bound on traceability rather than as a sentence-level factuality measure, and we discuss this limitation explicitly in Section 7. This formulation captures whether each cluster-level interpretation can be traced back to a properly formatted external identifier; it does not, by itself, certify that the cited identifier is the most appropriate reference for the specific biological claim, nor does it constitute a manual factual audit. We therefore use the term “ungrounded identifier output” or “non-verifiable identifier output” interchangeably with “hallucination” throughout, and we treat citation correctness and biological relevance as separate evaluative dimensions discussed in Section 7 and assessed qualitatively through the case studies in Section 6.5. To avoid false positives from generic numeric strings or mention of database names without valid identifiers, verification required explicit identifier-formatted matches rather than loose keyword detection. In the final multi-dataset analysis, this metric is the primary identifier-level robustness signal because it is available consistently across datasets and directly reflects source-traceable grounding.

#### 5.3.5 Evidence tier assignment

In addition to continuous metrics, BIOGEN assigns each interpretation to one of three evidence tiers derived from the empirical distribution of consensus critic scores. Thresholds are defined at the 33rd and 66th percentiles of all cluster scores, producing three data-driven categories: High confidence, Moderate confidence, and Suggestive. This tiering scheme provides an interpretable summary of evidential support without relying on manually selected cutoffs. Because the thresholds are derived independently within each dataset run, evidence-tier counts should be interpreted as relative within-run summaries rather than absolute cross-dataset confidence measures.

### 5.4 Framework configurations

This subsection describes the controlled settings used for the agentic framework comparison reported later in the Results section. In the final comparison, all five evaluated systems, including BIOGEN, were tested across the same five RNA-seq datasets: the primary S. enterica cohort (PRJEB67574) and four external bacterial datasets (GSE251671, GSE144604, GSE224463, and GSE55197). For every framework and dataset, we used the same LLM backbone (Mistral-7B-Instruct-v0.2, 4-bit NF4 quantization), the same evidence sources (PubMed and UniProt), and the same cluster inputs. Agentic orchestration was therefore the main experimental variable across systems.

To maintain comparability, all baseline systems used the same shared retrieval utilities and the same evidence pool construction policy as BIOGEN. Consequently, differences in output quality are attributable primarily to framework behavior and orchestration strategy rather than differences in evidence access or backbone model selection.

The four open-source baseline systems were configured as follows. LangChain ReAct (Yao et al., 2022) used a single ReAct-style reasoning loop over the shared evidence context. CrewAI used a sequential two-agent design consisting of a Retriever-style evidence consumer and an Interpreter-style reasoning agent. AutoGen (Wu et al., 2024; Dibia et al., 2024) used a two-turn orchestration pattern between an Orchestrator agent and a BioAssistant agent with the same retrieved evidence injected into context. Smolagents (Roucher et al., 2025) used a lightweight tool-calling reasoning loop over the shared evidence inputs.

We also considered comparison against domain-specific biomedical agent systems reported in the literature, including Med-PaLM (Singhal et al., 2023), the biomedical-domain language model BioGPT (Luo et al., 2022), the medical-domain pretrained model collection BioMistral (Labrak et al., 2024), and the multi-agent biomedical reasoning framework MedAgents (Tang et al., 2024). However, some of these systems depend on proprietary APIs or partially closed inference pipelines, while others were not directly applicable to the gene-cluster-interpretation task without nontrivial adaptation, which makes strict reproduction under the same local and controlled environment difficult. For this reason, the present comparison focuses on open-source frameworks that could be evaluated under a shared and reproducible setup; the systems above are listed for context to make the broader landscape visible to readers, even though they were not included in the controlled comparison of Section 6.6. Extending the benchmark to biomedical agent systems with comparable public implementations remains an important direction for future work.

In addition to the agentic framework comparison, controlled variants of retrieval-source selection and evidence-tier thresholding were used in the design-sensitivity analyses reported in Section 6.2.

## 6 Results

This section presents the empirical evaluation of BIOGEN across five complementary dimensions. We first examine grounding and reliability on the primary S. enterica dataset through system ablations, evidence-grounding metrics, critic analysis, and qualitative output comparison. We then present targeted design-sensitivity analyses for key architectural choices, assess complementarity with standard enrichment analyses, evaluate generalization across additional bacterial RNA-seq datasets, and finally compare BIOGEN with representative open-source agentic AI frameworks under a controlled setting.

### 6.1 Grounding and reliability on the primary dataset

**FIGURE 2:**
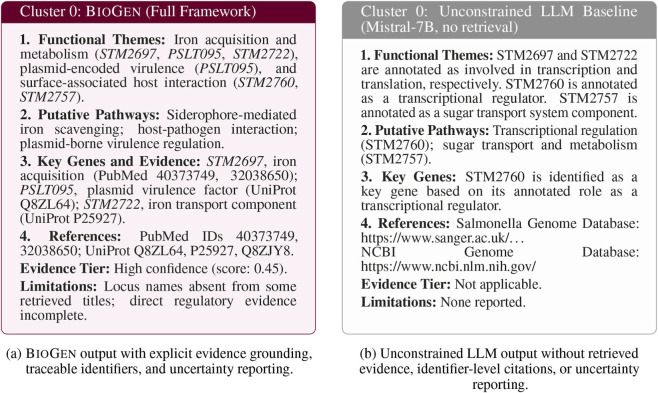
Qualitative comparison of BIOGEN and an unconstrained LLM baseline for Cluster 0 (*Salmonella enterica*). Both systems use the same LLM backbone, Mistral-7B-Instruct-v0.2. The baseline operates without external retrieval or verification, whereas BIOGEN incorporates retrieval, structured synthesis, and critic-based validation. As illustrated here, BIOGEN reports source-specific identifiers, assigns an evidence tier, and notes limitations, while the baseline produces plausible but non-traceable claims supported only by generic database links. This figure complements the quantitative comparison by showing how differences in grounding and traceability appear in an individual cluster-level output. For the BIOGEN Cluster 0 example shown in panel (a), the per-cluster BERTScore ( ${\text{F}}_{1}$ ) is 0.704 and the per-cluster Semantic Alignment Score is 0.520, computed against the same retrieval-pool reference text used in the dataset-level metrics; the full per-cluster distribution for the primary dataset is given in Supplementary Appendix Table 16.

We first evaluated BIOGEN on the primary *S. enterica* dataset to examine whether retrieval and critic-based verification improve grounding and interpretive reliability. Table 1 reports the effect of progressively adding retrieval and critic modules, Table 2 presents the controlled evidence-grounding comparison across matched system configurations, Table 3 analyzes the contribution of individual critic components, and Figure 2 provides an illustrative qualitative comparison between BIOGEN and an unconstrained LLM baseline.

**TABLE 1:** System-level ablation examining the impact of retrieval and critic modules on the identifier-based hallucination rate (i.e., the proportion of interpretations lacking a verifiable PMID, DOI, or UniProt accession; Section 5.3.4).

Configuration	Retriever	Critics	Identifier-based hallucination ↓
LLM only (Mistral-7B)	×	×	0.100
+ Retrieval	✓	×	0.000
+ Critics (Full BIOGEN)	✓	✓	**0.000**

The symbol × indicates that the corresponding component is absent, whereas ✓ indicates that it is enabled. Row labels with a leading “+” denote cumulative addition of components relative to the preceding configuration. Retrieval eliminates non-verifiable identifier outputs under the strict identifier-based criterion, while the critic ensemble further constrains grounded interpretations through post-generation verification. Bold values indicate the best-performing result for the corresponding evaluation metric.

**TABLE 2:** Controlled evidence-grounding comparison across system configurations on the primary *Salmonella enterica* dataset.

System	BERTScore ↑	SAS ↑	KEGG Sim. ↑	Identifier-based hallucination ↓
LLM only	–	–	–	0.100
LLM + retrieval	0.686	0.686	0.323	0.000
SimpleRAG	0.701	0.715	0.301	0.000
BIOGEN (full framework)	0.689	0.715	0.342	0.000

LLM + Retrieval uses the same retriever and LLM backbone as BIOGEN without critic-based verification. SimpleRAG is a strong single-agent retrieval-grounded baseline using the same evidence sources and representative genes but no critic ensemble. The symbols ↑ and ↓ indicate that higher and lower values are better, respectively. Dashes indicate that the metric is undefined for the LLM-only setting because no retrieved evidence is available.

**TABLE 3:** Critic-level ablation. Each configuration removes one critic module while holding all other components fixed.

Configuration	BERTScore ↑	SAS ↑	Identifier-based hallucination ↓
All critics (full)	0.723	0.600	0.000
−Semantic	0.737	0.633	0.000
−Adversarial	0.733	0.618	0.000
−Evidence-strict	0.735	**0.662**	0.000

A leading “−” in the configuration name indicates that the corresponding critic is omitted from the full BIOGEN setup. The symbols ↑ and ↓ indicate that higher and lower values are better, respectively. All configurations produced zero non-verifiable identifier outputs under the strict identifier-based criterion, because retrieval remains the primary grounding mechanism, while metric variation reflects each critic’s contribution to filtering stringency. Bold values indicate the best-performing result for the corresponding evaluation metric.

#### 6.1.1 Effect of retrieval and verification

Table 1 shows that retrieval is the main mechanism underlying the reduction in non-verifiable identifier outputs in the controlled primary-dataset setting. The matched LLM-only baseline produced an identifier-based hallucination rate of 0.10, indicating that unsupported or non-verifiable outputs still occurred when interpretation relied solely on parametric model knowledge. Once PubMed and UniProt retrieval were introduced, this rate dropped to 0.00 across all clusters under the strict identifier-based criterion. Adding the critic ensemble did not further reduce this metric, but it introduced an additional layer of filtering that improved claim-level grounding and biological coherence, as examined in Table 2.

These results indicate that external retrieval is necessary for traceable evidence grounding, whereas critic-based verification functions primarily as a reliability-control layer over already grounded outputs rather than as the sole mechanism for eliminating non-verifiable identifier outputs.

#### 6.1.2 Evidence-grounding quality

Table 2 summarizes the controlled comparison across four system configurations on the primary Salmonella enterica dataset: LLM only, LLM + Retrieval, SimpleRAG, and the full BIOGEN framework. SimpleRAG is a strong single-agent retrieval-grounded baseline introduced here for the first time: it uses the same LLM backbone, the same per-cluster representative genes, and the same PubMed and UniProt evidence sources as BIOGEN, but produces a cluster interpretation in a single LLM call without the critic ensemble or the two-pass evidence-tier assignment. SimpleRAG isolates the contribution of retrieval and structured prompting from the contribution of critic-based verification, and is described in full in Supplementary Appendix B. The LLM-only setting is not comparable on retrieval-conditioned metrics because it does not operate over an evidence pool, but it serves as a useful reference for identifier-level grounding behavior.

Among retrieval-grounded systems, LLM + Retrieval achieved a BERTScore of 0.686, SAS of 0.686, and KEGG Functional Similarity of 0.323, while producing zero non-verifiable identifier outputs. The stronger SimpleRAG baseline improved semantic alignment further, reaching 0.701 BERTScore and 0.715 SAS, although KEGG Functional Similarity remained lower at 0.301. The full BIOGEN framework also produced zero non-verifiable identifier outputs, matched the highest SAS value (0.715), and achieved the strongest KEGG Functional Similarity at 0.342.

Taken together, these results suggest a staged pattern. Retrieval provides the main factual grounding benefit; a strong single-agent RAG baseline further improves semantic alignment; and the critic-verified BIOGEN pipeline preserves traceability while improving biological coherence.

#### 6.1.3 Contribution of the critic ensemble

Table 3 examines the effect of removing individual critic modules while holding all other components fixed. All critic configurations produced an identifier-based hallucination rate of 0.00 under the strict identifier-based criterion, which is consistent with the earlier observation that retrieval is the main driver of factual grounding. At the same time, differences emerged in BERTScore and SAS, indicating that the critic modules influence output selection in distinct ways.

In particular, removing the Evidence-Strict critic produced the highest SAS (0.662), suggesting that this critic imposes the strongest constraint on which interpretations are retained. Removing the Semantic or Adversarial critics also altered the metric balance, although to a lesser extent. Overall, the critic ensemble appears to operate as a graded reliability mechanism rather than as a simple binary filter, with each component contributing a distinct evaluative perspective.

#### 6.1.4 Qualitative comparison with an unconstrained baseline

Figure 2 presents a qualitative comparison between BIOGEN and an unconstrained LLM baseline on the same gene cluster (Cluster 0, Salmonella enterica, ENA: PRJEB67574). The unconstrained baseline uses the same LLM backbone, Mistral-7B-Instruct-v0.2, but operates without retrieval, external evidence, or critic-based verification. This setting corresponds to the LLM-only configuration reported in Table 1. BIOGEN analyzes the same cluster using the full pipeline, including PubMed and UniProt retrieval, structured interpretation, and multi-critic validation.

The comparison highlights several practical differences between evidence-grounded interpretation and unconstrained generation. BIOGEN reports verifiable PubMed identifiers and UniProt accessions, enabling direct source traceability. It also links gene-level claims to retrieved evidence and explicitly reports both evidence tier and limitations. By contrast, the unconstrained baseline produces plausible biological text, but without article-level or protein-level identifiers and without explicit acknowledgment of evidential gaps. Figure 2 therefore complements the quantitative results by illustrating how differences in grounding and traceability appear in an individual cluster-level output.

Cluster 0: BIOGEN (Full Framework) 1. Functional Themes: Iron acquisition and metabolism (*STM2697*, *PSLT095*, *STM2722*), plasmid-encoded virulence (*PSLT095*), and surface-associated host interaction (*STM2760*, *STM2757*). 2. Putative Pathways: Siderophore-mediated iron scavenging; host-pathogen interaction; plasmid-borne virulence regulation. 3. Key Genes and Evidence: *STM2697*, iron acquisition (PubMed 40373749, 32038650); *PSLT095*, plasmid virulence factor (UniProt Q8ZL64); *STM2722*, iron transport component (UniProt P25927). 4. References: PubMed IDs 40373749, 32038650; UniProt Q8ZL64, P25927, Q8ZJY8. Evidence Tier: High confidence (score: 0.45). Limitations: Locus names absent from some retrieved titles; direct regulatory evidence incomplete. (a) BIOGEN output with explicit evidence grounding, traceable identifiers, and uncertainty reporting.

Cluster 0: Unconstrained LLM Baseline (Mistral-7B, no retrieval) 1. Functional Themes: STM2697 and STM2722 are annotated as involved in transcription and translation, respectively. STM2760 is annotated as a transcriptional regulator. STM2757 is annotated as a sugar transport system component. 2. Putative Pathways: Transcriptional regulation (STM2760); sugar transport and metabolism (STM2757). 3. Key Genes: STM2760 is identified as a key gene based on its annotated role as a transcriptional regulator. 4. References: *Salmonella* Genome Database: https://www.sanger.ac.uk/… NCBI Genome Database: https://www.ncbi.nlm.nih.gov/Evidence Tier: Not applicable. Limitations: None reported. (b) Unconstrained LLM output without retrieved evidence, identifier-level citations, or uncertainty reporting.

### 6.2 Design sensitivity analyses

To further examine key design choices in BIOGEN, we conducted three targeted analyses on the primary *S. enterica* dataset. These analyses focus on retrieval source selection, the stability of percentile-based evidence tiers, and the specificity of retrieved evidence at the gene level. Together, they provide additional context for understanding how the framework behaves under its current design.

#### 6.2.1 Retrieval source ablation

We first examined whether the choice of retrieval source alone changes the quality of the generated interpretations. The BIOGEN pipeline was run under three source configurations: PubMed only, UniProt only, and PubMed plus UniProt, which is the default setting used by the full framework. All configurations used the same clustering outputs, gene selection procedure, and LLM backbone. To isolate the effect of source selection itself, this analysis was performed without the critic ensemble. Table 4 reports BERTScore, SAS, and the identifier-based hallucination rate across the three settings.

**TABLE 4:** Retrieval source ablation on the primary S. enterica dataset.

Retrieval source	BERTScore ↑	SAS ↑	Identifier-based hallucination ↓
PubMed only	0.674	0.035	1.000
UniProt only	0.674	0.035	1.000
PubMed + UniProt (default)	0.674	0.035	1.000

All configurations use the same clustering outputs and LLM backbone. Metrics are computed without the BIOGEN critic ensemble in order to isolate the effect of source selection on raw generation behavior. The symbols ↑ and ↓ indicate that higher and lower values are better, respectively.

The three configurations produced identical values under this critic-free setting. This indicates that retrieval source selection alone was not sufficient to improve grounding in the absence of explicit verification. In all three cases, the model generated biologically plausible text, but the outputs did not contain verifiable identifier-level support, resulting in an identifier-based hallucination rate of 1.000. When read together with Table 1, this result reinforces the view that retrieval alone provides evidence context, whereas the critic ensemble is the component that converts that context into traceable, reporting-constrained output.

Numerically identical BERTScore and SAS values across the three retrieval configurations may at first appear surprising. Two factors account for this. First, in all three settings the LLM produced free-form biological text without inline identifiers, so the lexical content of the generated interpretations was driven primarily by the LLM’s parametric knowledge rather than by which retrieval source was attached to the prompt; under those conditions, BERTScore and SAS, which compare the interpretation against the same shared evidence reference pool, produce closely overlapping values that round to the same three decimal places at the run-aggregated level. Second, the critic-free retrieval-only setting does not enforce that the LLM cite or even read the retrieved evidence; without that constraint, source-specific differences fail to propagate into the generated text. The three-way agreement should therefore be read as a property of the critic-free regime, not as evidence that the two retrieval sources are interchangeable in the full BIOGEN pipeline. The default use of both PubMed and UniProt is motivated by source complementarity within the full framework rather than by differences in unconstrained generation alone: PubMed contributes abstract-level literature context and PMID-based provenance, whereas UniProt contributes structured accession-linked protein annotations. In the full BIOGEN setting, these sources provide complementary anchors for critic-based validation and final reporting.

#### 6.2.2 Evidence tier threshold sensitivity

BIOGEN assigns evidence tiers using percentile-based thresholds derived from the consensus score distribution, with the default setting based on the 33rd and 66th percentiles. To examine whether this choice is sensitive to the exact threshold values, we reassigned evidence tiers under three percentile schemes: p25/p75, p33/p66, and p40/p60. Table 5 summarizes the resulting tier distributions and inter-configuration agreement.

**TABLE 5:** Evidence tier threshold sensitivity analysis on the primary *S. enterica* dataset.

Config	Lower	Upper	High	Moderate	Suggestive	κ
p25/p75	0.446	0.450	7	0	3	1.000
p33/p66 (default)	0.450	0.450	7	0	3	
p40/p60	0.450	0.450	7	0	3	

Tier labels are reassigned under three percentile-based threshold schemes using the same consensus scores. κ denotes pairwise agreement across threshold variants.

Tier assignments were unchanged across all three threshold settings, with perfect agreement (κ=1.000) . In this dataset, the consensus scores occupy a relatively narrow range, so nearby percentile choices produce nearly identical decision boundaries. This result indicates that the default p33/p66 scheme is stable under modest threshold variation and does not appear to be sensitive to small changes in percentile selection.

#### 6.2.3 Evidence specificity analysis

To characterize the nature of the retrieved evidence, we computed an Evidence Specificity Score (ESP) for each gene in the primary dataset. ESP measures the proportion of retrieved records that explicitly mention a gene’s locus tag, symbol, or protein name within the retrieved text. Across 372 evidence records spanning all 10 clusters, the mean ESP was 0.003, and 99.7% of records had ESP = 0.000.

These values indicate that the retrieved evidence was dominated by organism-level or pathway-level context rather than direct gene-specific discussion. This pattern is biologically plausible for Salmonella LT2 locus tags, which are often not mentioned explicitly in titles or abstracts even when the surrounding biological process is relevant. The low ESP distribution therefore helps contextualize two observed features of the primary-dataset results: the conservative evidence-tier profile and the moderate KEGG Functional Similarity score. Under sparse gene-specific citation support, BIOGEN tends to report broader mechanistic context while explicitly signaling evidential limitations when direct support is weak.

More broadly, the ESP analysis suggests that the difficulty of this interpretive setting arises not only from model behavior, but also from the specificity of the available literature itself. In such cases, BIOGEN’s value lies in organizing partial and heterogeneous evidence into structured explanations while preserving explicit uncertainty cues, rather than presenting unsupported gene-level assertions as established fact.

### 6.3 Complementarity with KEGG enrichment on the primary dataset

We next compared BIOGEN with a KEGG over-representation analysis baseline on the primary *Salmonella enterica* dataset to examine whether literature-grounded interpretation provides biological context beyond pathway statistics alone. Table 6 reports the significant KEGG pathways identified for this dataset, and Table 7 quantifies the overlap between BIOGEN-derived themes and KEGG/ORA outputs across all 10 clusters. A broader comparison with standard enrichment baselines across datasets, including GO/ORA where annotation coverage permits, is presented later in Section 6.4.

**TABLE 6:** KEGG over-representation analysis results per cluster (Fisher’s exact test, BH-corrected adj. p<0.05 ). Organism: *Salmonella enterica* Typhimurium LT2 (KEGG code: stm). Only clusters with at least one significant term are shown.

Cluster	Top KEGG pathway	Overlap	Adj. p
C7	Bacterial secretion system	3/38	$5.71\times 10^{-5}$
C7	Two-component system	2/61	$3.2\times 10^{-2}$
C8	Membrane transport	2/44	$4.1\times 10^{-2}$
C8	Metabolic pathways	3/189	$4.8\times 10^{-2}$
C0–C6, C9	(No significant terms)	–	–

**TABLE 7:** Complementarity analysis between BIOGEN and KEGG/ORA across 10 gene clusters.

Cluster	BIOGEN	KEGG/ORA	Shared	BIOGEN-unique	Jaccard
C0	8	0	0	8	0.000
C1	7	0	0	7	0.000
C2	8	1	1	7	0.125
C3	5	0	0	5	0.000
C4	6	0	0	6	0.000
C5	10	0	0	10	0.000
C6	8	0	0	8	0.000
C7	8	2	1	7	0.111
C8	8	2	2	6	0.250
C9	6	0	0	6	0.000
Mean	7.4	0.5	0.4	7.0	0.049

BIOGEN-unique themes represent mechanistic context recoverable only through literature-grounded reasoning. Jaccard index measures thematic overlap between the two methods. Note: the KEGG/ORA column reports themes derived by keyword-mapping the KEGG term names returned by the enrichment analysis to the same biological-theme taxonomy used to extract themes from BIOGEN interpretations (see implementation in kegg_ora_overlap_analysis.py). The KEGG/ORA-theme counts in this table can therefore include themes recovered from KEGG terms that did not pass the strict adj. p<0.05 threshold reported in Table 6; for example, Cluster two yields one mapped theme here even though it does not appear among the FDR-significant clusters in Table 6. The two tables are therefore consistent under their respective definitions but use different inclusion criteria for what counts as a KEGG/ORA “term”.

Table 6 shows that only two of the 10 clusters produced statistically significant KEGG pathway associations after multiple-testing correction. Specifically, Cluster seven was associated with the bacterial secretion system and the two-component signaling system, whereas Cluster eight showed enrichment for membrane transport and metabolic pathways. The remaining 8 clusters did not yield any significant KEGG terms.

Table 7 shows that BIOGEN identified a mean of 7.4 biological themes per cluster, whereas KEGG/ORA identified 0.5. Across the 10 clusters, BIOGEN produced 70 unique biological themes not recovered by enrichment analysis, whereas enrichment contributed only 1 theme not captured by BIOGEN. The mean Jaccard index of 0.049 indicates limited thematic overlap between the two approaches.

This low overlap should not be interpreted as a weakness of either method. Rather, it reflects a structural difference between pathway-level enrichment and literature-grounded interpretation. KEGG/ORA is effective when cluster genes align with curated pathway sets, but it provides limited coverage when gene sets are small, diffuse, or poorly represented in existing databases. BIOGEN, by contrast, can recover mechanistic context, regulatory associations, stress-response links, and host-pathogen interaction themes that are not explicitly encoded as pathway membership. In this sense, the two approaches provide complementary views of the same transcriptomic structure rather than interchangeable outputs.

### 6.4 Generalization beyond the primary dataset

To examine whether BIOGEN generalizes beyond the primary *Salmonella enterica* cohort, we evaluated the same pipeline on four additional bacterial RNA-seq datasets spanning *Escherichia coli* and *Pseudomonas aeruginosa*. These datasets were selected to introduce variation in organism, experimental setting, and transcriptional context while remaining relevant to antimicrobial resistance and bacterial stress-response biology. No organism-specific modifications were introduced to the retrieval module, critic ensemble, language model backbone, or clustering procedure.

The complete evaluation set therefore consisted of five datasets:

- *S. enterica* Typhimurium LT2 (ENA: PRJEB67574) – 58 samples, 4,679 genes, primary dataset.
- *P. aeruginosa* UCBPP-PA14 (GEO: GSE251671) – large-scale antibiotic perturbation and CRISPRi transcriptional profiling.
- *E. coli* MG1655 (GEO: GSE144604) – transcriptomic responses to antibiotic and biocide combinations.
- *E. coli* K-12 (GEO: GSE224463) – multi-condition stress-response time-course transcriptomics.
- *P. aeruginosa* PA14 (GEO: GSE55197) – transcriptional responses across biofilm, planktonic, and environmental growth conditions.

Across all datasets, we kept fixed the clustering configuration ( k=10 , gene-level KMeans), evidence retrieval sources (PubMed and UniProt), language model backbone (Mistral-7B-Instruct-v0.2, 4-bit NF4), and the three-critic evaluation framework. We treat the primary *Salmonella* dataset as an in-domain reference setting and the remaining datasets as external distribution shifts that vary by organism, condition, and evidence density.

#### 6.4.1 Evidence grounding under distribution shift

Table 8 summarizes BIOGEN performance across all five datasets under the fixed pipeline configuration. Across all evaluated settings, the identifier-based hallucination rate remained 0.000, indicating that every BIOGEN interpretation contained at least one verifiable PMID, DOI, or UniProt accession from the per-dataset retrieval pool. This pattern was the most stable identifier-level robustness signal in the multi-dataset evaluation; we emphasize, however, that it does not on its own certify that each cited identifier is the most appropriate reference for every gene-level claim, a point we return to in Section 7 and in the case studies of Section 6.5.

**TABLE 8:** BIOGEN under distribution shift. All datasets were processed using the same clustering procedure, retrieval sources, LLM backbone, and critic ensemble.

Dataset	Shift type	BERTScore ↑	SAS ↑	KEGG sim ↑	Identifier-based hallucination ↓	Mean critic score
PRJEB67574	in_domain	0.689	0.715	0.342	0.000	0.446
GSE144604	cross_organism	0.715	0.504	N/A	0.000	0.353
GSE55197	cross_organism	0.699	0.195	N/A	0.000	0.387
GSE251671	cross_organism	0.561	−0.016	N/A	0.000	0.243
GSE224463	cross_condition	0.550	−0.003	0.154	0.000	0.320

The symbols ↑ and ↓ indicate that higher and lower values are better, respectively. N/A indicates that the corresponding metric was not computed because of insufficient coverage or unavailable evaluation support.

BERTScore values ranged from 0.550 to 0.715, showing that semantic correspondence between generated interpretations and retrieved evidence remained broadly stable across organisms. The highest BERTScore was observed for GSE144604 (0.715), followed by GSE55197 (0.699) and the primary S. enterica dataset (0.689). Lower values were obtained for GSE251671 (0.561) and GSE224463 (0.550), suggesting that some external datasets provide weaker or less specific literature support for the clustered genes.

Semantic Alignment Score (SAS) varied more substantially across datasets. Stronger alignment was observed on the primary dataset and GSE144604, whereas other external datasets showed near-zero or slightly negative values. Because the retrieved evidence pools differ in density and specificity across organisms, SAS should be interpreted cautiously in cross-dataset settings and is best viewed as a relative within-dataset alignment signal rather than a uniform robustness metric. KEGG Functional Similarity could be computed only when sufficient overlap existed between cluster genes and organism-specific KEGG annotations. Accordingly, the strongest cross-dataset conclusion is anchored on identifier-based source traceability and the preservation of evidence-grounded interpretation under shift.

#### 6.4.2 Evidence-tier robustness across datasets

In addition to continuous metrics, we examined how BIOGEN’s evidence-tier assignments behaved across datasets. Table 9 summarizes the distribution of High confidence, Moderate confidence, and Suggestive interpretations for each run. The primary dataset produced a 7/0/3 split, whereas the external cohorts showed broader variation, including a 10/0/0 profile for GSE144604 and a more mixed 4/3/3 profile for GSE251671.

**TABLE 9:** Evidence-tier robustness across datasets. Tier counts are relative within each dataset run and should not be interpreted as absolute cross-dataset confidence levels.

Dataset	Shift	High	Moderate	Suggestive	Mean score
PRJEB67574	in_domain	7	0	3	0.446
GSE144604	cross_organism	10	0	0	0.353
GSE55197	cross_organism	6	1	3	0.387
GSE251671	cross_organism	4	3	3	0.243
GSE224463	cross_condition	7	0	3	0.320

These differences should not be interpreted as absolute cross-dataset confidence rankings. Evidence tiers are assigned from percentile-based thresholds derived independently within each run, so they are relative to the score distribution of the dataset under analysis. Even so, the table is informative as a compact summary of how critic consensus varies under different shift settings. Notably, all datasets still retained multiple within-run high-confidence outputs while producing zero non-verifiable identifier outputs under the strict identifier-based criterion.

#### 6.4.3 Complementarity with standard enrichment methods across organisms

Table 10 compares BIOGEN with two standard enrichment baselines, KEGG/ORA and GO/ORA, across the available datasets. Both enrichment analyses use Fisher’s exact test with Benjamini–Hochberg FDR correction (α=0.05) and identical background gene sets, thereby supporting a consistent comparison.

**TABLE 10:** Cross-organism comparison between BIOGEN and two standard enrichment baselines, KEGG/ORA and GO/ORA.

Dataset	Organism	BIOGEN	KEGG	GO	Sig. clusters (KEGG)	Jaccard	Ratio
*Primary dataset*							
PRJEB67574	*S. enterica*	7.4	0.5	0.0	2/10	0.049	14.8×
*Cross-organism validation*							
GSE251671	*P. aeruginosa* PA14	13.2	0.0	–	0/10	0.000	∞
GSE144604	*E. coli* MG1655	17.0	0.0	–	0/10	0.000	∞
GSE224463	*E. coli* K-12	13.1	0.7	–	4/10	0.054	18.7×
GSE55197	*P. aeruginosa* PA14	7.6	0.1	–	1/10	0.025	76.0×

Both enrichment analyses use Fisher’s exact test with Benjamini–Hochberg FDR correction (α=0.05) and identical background gene sets. “Themes/cluster” denotes the mean number of biological themes or significant enrichment terms identified per gene cluster. “Sig. clusters (KEGG)” reports the number of clusters with at least one significant KEGG pathway. The symbol ∞ indicates datasets for which the enrichment baseline returned zero significant terms across all clusters. GO/ORA was evaluated only on the primary *S. enterica*dataset; “–” denotes datasets for which GO/ORA was not run because of annotation coverage limitations for bacterial locus tags.

Across all evaluated datasets, BIOGEN recovered substantially more cluster-level biological themes than either enrichment method. BIOGEN identified an average of 7.4–17.0 themes per cluster, whereas KEGG/ORA returned between 0.0 and 0.7 significant terms per cluster. For the primary *S. enterica* dataset, GO/ORA returned no significant terms across any cluster.

The GO/ORA null result on the primary dataset is informative in itself. It is consistent with the sparse KEGG/ORA signal on the same dataset (2/10 significant clusters) and with the low Evidence Specificity Score distribution reported in Section 6.2. Taken together, these observations suggest that *S. enterica* LT2 STM locus tags have limited direct coverage in standard annotation resources. Because pathway- and ontology-based enrichment depend on this annotation layer, they provide sparse output for most clusters in this setting. By contrast, BIOGEN is not restricted to database membership alone and instead retrieves supporting primary literature for individual genes, which allows it to recover broader biological themes even when structured enrichment returns little or no signal.

The contrast was especially pronounced for GSE251671 and GSE144604, where KEGG/ORA returned no significant pathways for any cluster while BIOGEN still recovered 13.2 and 17.0 themes per cluster, respectively. For GSE224463, where KEGG/ORA identified significant pathways in four of 10 clusters, BIOGEN still provided substantially broader thematic coverage at 13.1 themes per cluster. The low Jaccard overlap observed across datasets indicates that BIOGEN and enrichment analysis capture different aspects of biological organization rather than redundant signal.

Overall, these findings suggest that the complementarity between BIOGEN and enrichment analysis is not limited to a single dataset or a single annotation source. The broader coverage of BIOGEN remains visible when compared with both pathway-level enrichment (KEGG/ORA) and ontology-level enrichment (GO/ORA), although GO/ORA could be evaluated only on the primary dataset because of annotation coverage limitations in the external bacterial cohorts.

### 6.5 Biological case studies

The metric-based evaluation in the previous subsections summarizes BIOGEN behavior across many clusters and datasets, but it does not by itself show how the framework behaves on individual gene clusters. To make this concrete, we present three complementary case studies drawn from the primary S. enterica dataset (PRJEB67574). The cases were chosen to span the range of regimes the framework is expected to encounter in practice: (i) a cluster where literature-grounded interpretation agrees with conventional KEGG over-representation analysis, (ii) a cluster where KEGG/ORA returns no significant terms but BIOGEN still produces a structured biological interpretation grounded in retrieved evidence, and (iii) a low-confidence cluster where the framework produces a conservative interpretation and explicitly reports limitations. The case studies are intended as illustrative examples of model behavior; they are not a substitute for the manual citation-correctness audit discussed in Section 7, which is flagged as planned future work.

Case 1: Recovery of a known mechanism (Cluster 7, KEGG-positive). For Cluster 7, KEGG/ORA identified the bacterial secretion system and the two-component system as significantly enriched (Table 6). Independently, the BIOGEN interpretation for the same cluster identifies several genes (gene-STM4489, gene-STM4526, gene-STM4495) as components of Salmonella pathogenicity island 1 (SPI-1), which encodes the type-III secretion system used for invasion of non-phagocytic host cells. The BIOGEN output cites supporting PubMed references and links these gene-level claims to the SPI-1 invasion phenotype, while also flagging a remaining gene (gene-PSLT108) as a putative transcriptional regulator whose specific regulatory role is not directly established in the retrieved evidence. This case provides an internal consistency check: where pathway enrichment yields a significant signal, BIOGEN’s literature-grounded interpretation arrives at a biologically compatible conclusion via a different evidence pathway, and additionally provides cluster-level mechanistic context (specific SPI-1 component genes, host-cell invasion phenotype) beyond what the KEGG term name alone reports.

Case 2: Mechanistic context where enrichment is silent (Cluster 5, KEGG-negative). Cluster five contains a high proportion of plasmid-encoded genes (PSLT068, PSLT011, PSLT044, PSLT051, PSLT052) together with chromosomal genes including STM1332. KEGG/ORA returned no significant pathway terms for this cluster after FDR correction, in agreement with the broader pattern in Table 6 that eight of 10 primary clusters yield no significant KEGG terms. BIOGEN nevertheless produces a structured interpretation that organizes the cluster around plasmid-borne Salmonella virulence and genomic variation, identifies PSLT068 as a putative transcriptional regulator implicated in pathogenicity, and notes STM1332 as a putative outer-membrane protein. The interpretation cites PubMed identifiers in the retrieval pool and explicitly states which gene-level claims are gene-specific versus organism-level generic, with a Limitations field acknowledging that “the provided literature does not explicitly state the specific functions and interactions among the genes in the identified gene cluster.” This case illustrates how BIOGEN can recover plausible mechanistic context for clusters that fall outside the reach of curated pathway annotations, while preserving uncertainty about the strength of the underlying evidence.

Case 3: A low-confidence, conservative interpretation (Cluster 3). Cluster three in this run contained a single representative gene (gene-STM4261) and received the lowest consensus critic score in the run. The BIOGEN interpretation for this cluster cites a single *Salmonella* genome variation reference (PMID: 29784861) and explicitly states “the precise functions of the genes within this cluster are not explicitly stated in the provided references,” “no specific pathways are mentioned in the provided evidence,” and “no information regarding transcriptional regulation of the STM4261 gene cluster was found in the given references.” The Limitations field reiterates that “the provided references do not offer sufficient information to determine the specific functions, biological pathways, or transcriptional regulation of the STM4261 gene cluster.” This case is included as a negative example: when retrieved evidence is sparse, BIOGEN does not generate confident mechanistic claims and instead returns a conservative interpretation flagged with a low consensus score and explicit evidential gaps. We view this behavior as a desirable property of an evidence-grounded interpretive layer, but we emphasize that it is also a direct consequence of how the framework was designed and prompted, not a guarantee under all conditions.

Together, these three cases illustrate the spectrum of behavior summarized quantitatively in the metric tables: agreement with enrichment where enrichment has signal, mechanistic context where enrichment is silent, and conservative reporting under low evidence support. They are illustrative rather than evaluative; a systematic expert-curated assessment of citation correctness and biological relevance is described in Section 7 as planned future work.

### 6.6 Comparison with agentic AI frameworks

To place BIOGEN within the broader landscape of agentic AI systems, we compared it with four representative open-source agent frameworks across the same five RNA-seq datasets used in the cross-organism evaluation. The goal of this experiment was to examine whether BIOGEN’s evidence-grounded behavior remains robust under distribution shift when compared with widely used orchestration strategies under a controlled setting. All systems used the same LLM backbone, the same evidence sources, and the same cluster inputs, so the main experimental variable was the agentic orchestration framework.

Controlled experimental conditions across baselines. To support a fair comparison, all four agentic baselines were run under shared experimental controls so that orchestration architecture remained the only intentional variable. Specifically: (i) all baselines and BIOGEN used the same Mistral-7B-Instruct-v0.2 backbone loaded by a shared model-loading utility with the same 4-bit NF4 quantization configuration, the same generation temperature (0.2), and the same maximum new-token budget; (ii) all baselines received the same per-dataset evidence pool, produced by BIOGEN’s retriever, injected into baseline prompts so that retrieval is not a confound; (iii) all systems were evaluated against the same cluster inputs (the same gene-level partitions and per-cluster representative gene lists from Experiment 1) and the same evaluation pipeline (the same BERTScore model, the same Sentence-BERT encoder for SAS, and the same identifier-based hallucination regex). Each baseline retained its framework-specific prompt structure–ReAct trace, two-role retriever-then-interpreter, coordinator-and-specialist, and tool-observation–so that observed differences reflect orchestration behavior rather than evidence access, model selection, or evaluation setup. The full per-framework prompt templates and the shared loader configuration are documented in Supplementary Appendix G.

Table 11 summarizes the controlled multi-dataset comparison, while the full per-dataset, per-framework results are provided in Supplementary Appendix Table 13. The clearest and most consistent pattern is identifier-level grounding behavior: BIOGEN produced zero non-verifiable identifier outputs on all five datasets under the strict identifier-based criterion, whereas all baseline frameworks exhibited substantially higher non-verifiable identifier rates, ranging from 0.1 to 1.0 depending on the dataset and framework. This makes identifier-level traceability the most robust point of separation between BIOGEN and the generic agentic baselines.

**TABLE 11:** Summary of controlled multi-dataset framework comparison.

Dataset	BIOGEN BERT ↑	Best baseline BERT ↑	BIOGEN SAS ↑	Best baseline SAS ↑	BIOGEN Id-Hall. ↓	Best baseline Id-Hall. ↓
PRJEB67574	0.689	0.736	0.715	0.841	0.000	0.300
GSE251671	0.561	0.602	−0.016	0.053	0.000	0.300
GSE144604	0.715	0.729	0.504	0.545	0.000	0.100
GSE224463	0.550	0.636	−0.003	0.095	0.000	0.800
GSE55197	0.699	0.728	0.195	0.573	0.000	0.300

For each dataset, BIOGEN is compared with the strongest non-BIOGEN baseline for each metric. The symbols ↑ and ↓ indicate that higher and lower values are better, respectively. Id-Hall. denotes the identifier-based hallucination rate (Section 5.3.4).

By contrast, similarity-based metrics showed a different pattern. Across datasets, the generic baselines often achieved higher BERTScore and SAS values than BIOGEN. On the primary dataset, for example, the strongest baseline reached BERTScore 0.736 and SAS 0.841, whereas BIOGEN achieved 0.689 and 0.715, respectively. Similar patterns were observed on the external datasets, as shown in Table 11 and in the detailed appendix results (Supplementary Appendix Table 13). This indicates that semantic-overlap metrics alone do not fully capture evidential reliability: fluent or semantically aligned outputs may still fail to provide explicit, verifiable source grounding. In this setting, BIOGEN’s main advantage is therefore not universal dominance on all text-similarity metrics, but the consistent production of traceable, citation-grounded interpretations under the same retrieval and backbone conditions.

Table 12 summarizes this tradeoff compactly. Across the five datasets, BIOGEN was best on the identifier-based hallucination rate in all cases, whereas the strongest generic baseline outperformed BIOGEN on BERTScore and SAS in each dataset. This result reinforces a broader methodological point for biomedical AI: retrieval access alone is insufficient if the final interpretation does not preserve identifier-level traceability. Agentic systems intended for scientific use should therefore be evaluated not only by semantic similarity, but also by their ability to produce transparent, auditable, and explicitly grounded outputs.

**TABLE 12:** Win/loss summary for BIOGEN against the strongest non-BIOGEN baseline across the five datasets.

Metric	BIOGEN wins	Baseline wins	Ties
Identifier-based hallucination ↓	5	0	0
BERTScore ↑	0	5	0
SAS ↑	0	5	0

## 7 Discussion

This study frames RNA-seq cluster interpretation as an evidence-grounded reasoning problem rather than as unconstrained biological text generation. Across the primary *Salmonella enterica* dataset, the controlled comparison suggests a layered contribution from the framework components. Retrieval is the primary driver of the reduction in non-verifiable identifier outputs, lowering the identifier-based hallucination rate from 0.10 in the matched LLM-only baseline to 0.00 in all retrieval-grounded settings, including the SimpleRAG single-agent baseline. BIOGEN’s contribution beyond SimpleRAG is therefore not a further reduction in the identifier-based hallucination rate, but the addition of structured reliability assessment, evidence-tier assignment, explicit limitation reporting, and an auditable, source-traceable output format. SimpleRAG and BIOGEN achieve identical (zero) non-verifiable identifier rates and similar SAS values, while BIOGEN additionally produces critic-aggregated consensus scores and the within-run *High*/*Moderate*/*Suggestive* tier labels that SimpleRAG does not. We do not claim that BIOGEN universally outperforms SimpleRAG or any of the agentic-framework baselines on similarity metrics; the empirical advantage of the full framework is the structured-reliability and traceability layer rather than higher BERTScore or SAS. This division of labor is important because it distinguishes the source of factual grounding (retrieval) from the source of interpretive filtering and reporting (the critic ensemble).

A central finding of the study is the complementary relationship between BIOGEN and standard enrichment analysis. On the primary dataset, both KEGG/ORA and GO/ORA returned sparse structured annotations, whereas BIOGEN produced literature-grounded interpretations for all clusters and recovered substantially broader cluster-level thematic coverage. The low thematic overlap between these approaches does not indicate conflict. Instead, it suggests that they operate at different levels of biological organization. Enrichment analysis functions at the level of predefined pathway or ontology membership, whereas BIOGEN can incorporate mechanistic context, regulatory associations, and literature-supported functional links that are not explicitly represented in annotation databases.

Implications for the Research Community From a broader research perspective, this study suggests that transcriptomic interpretation should not be viewed only as a problem of statistical enrichment or only as a problem of fluent text generation. Between these two extremes lies an important intermediate task: producing biological explanations that are both source-traceable and usable for downstream scientific reasoning. More generally, the findings support a shift in how biomedical agent systems are evaluated. In addition to output quality, future work should ask whether such systems make their evidential basis explicit, preserve uncertainty when annotation is sparse, and remain auditable enough to support expert review rather than bypass it.

The multi-dataset analysis further suggests that BIOGEN’s grounding behavior remains stable beyond a single organism or experimental setting. Across additional *Escherichia coli* and *Pseudomonas aeruginosa* RNA-seq studies, BIOGEN produced zero non-verifiable identifier outputs under the strict identifier-based criterion across all evaluated distribution shifts. This result became the strongest and most stable cross-dataset identifier-level robustness signal in the study, although as discussed in the limitations below, it does not by itself certify the relevance or biological correctness of every cited identifier. At the same time, variation in BERTScore, SAS, and KEGG similarity across external datasets indicates that evidence density and annotation coverage remain important determinants of downstream interpretive quality. In particular, some datasets provided weaker or less specific literature support, which reduced semantic alignment even though the overall evidence-grounded generation process remained stable.

The framework comparison highlights a broader methodological lesson for biomedical AI. In the corrected multi-dataset comparison, generic agentic baselines often achieved higher similarity-based scores than BIOGEN, yet they consistently produced higher non-verifiable identifier rates and weaker identifier-level traceability. This distinction is important. In scientific interpretation tasks, semantic similarity alone is not sufficient if the final explanation cannot be explicitly connected to verifiable supporting sources. The main advantage of BIOGEN is therefore its reliable grounding behavior: under all evaluated distribution shifts, it remained the only framework to produce zero non-verifiable identifier outputs across all five datasets.

Several limitations should be noted. First, the current implementation focuses on PubMed and UniProt and therefore depends on the density and specificity of available external evidence for a given organism and gene set. This dependence introduces a structural bias toward well-studied genes: locus tags or proteins that are heavily indexed in PubMed accumulate gene-specific evidence quickly, whereas understudied or poorly annotated genes return mostly organism-level generic context. The Evidence Specificity Score analysis in Section 6.2 (mean ESP =0.003 across 372 records on the primary dataset) shows this concretely: most retrieved evidence on *S. enterica* LT2 STM locus tags is generic rather than directly gene-specific. The downstream consequence is that BIOGEN can recover broader mechanistic context for understudied clusters, but the strength of that context is upper-bounded by the indexing coverage of the underlying biomedical literature. Coverage of orphan or under-represented genes is therefore an inherent limitation rather than a tunable parameter, and it cannot be fully solved by changing r alone. Incorporating structured signature resources such as MSigDB or other organism-specific knowledge bases may improve evidence coverage and functional specificity. We additionally note that the identifier-based hallucination metric used throughout the paper measures whether each interpretation contains a properly formatted PMID, DOI, or UniProt accession from the retrieval pool. Citation presence does not, on its own, guarantee that the cited reference is the most appropriate source for a specific biological claim, nor that the claim itself is biologically correct. We therefore frame the metric as an identifier-level source-traceability signal rather than as a complete factual audit, and we plan a manual citation-correctness audit on a stratified sample of cluster–citation pairs as a follow-up analysis (see Section 8).

Second, not all evaluation metrics were available uniformly across datasets. In particular, some metrics were not computable in all external settings because of evidence coverage limitations or dataset-specific annotation constraints. For this reason, the strongest cross-dataset conclusions are anchored primarily on identifier-based source traceability, structured evidence reporting, and the preservation of grounded interpretation under shift. Third, evidence-tier labels (*High confidence*, *Moderate confidence*, *Suggestive*) are derived from percentile-based thresholds computed within each pipeline run. These tiers are dataset-relative ranks rather than absolute confidence levels: a “High confidence” interpretation in one dataset is not directly comparable to a “High confidence” interpretation in another. We chose this design because the consensus-score distribution shifts across datasets in ways that depend on evidence density, and a fixed cross-dataset threshold would be misleading. The cost of this design is that the labels do not provide absolute calibration. Producing dataset-independent absolute confidence scores would require an external reference standard (e.g., expert-rated cluster interpretations) that we do not yet have. Fourth, although the framework comparison includes multiple open-source agentic baselines, the present study does not include domain-specific biomedical agents that require proprietary or non-reproducible infrastructure. Fifth, the design choices in BIOGEN were motivated by interpretability and traceability considerations, but alternative orchestration strategies, such as earlier-stage evidence verification or critic-in-the-loop generation, remain worth exploring more systematically.

A practical limitation of the current framework is that retrieval-grounded multi-agent verification introduces additional computational overhead relative to single-pass baselines, with the main added cost arising from evidence retrieval and critic evaluation. In addition, the present study uses K-means as the upstream clustering method because it provides a simple, transparent, and reproducible way to generate gene modules for downstream interpretation. This choice was intended to isolate the contribution of BIOGEN at the interpretation stage rather than to optimize the clustering procedure itself. Because BIOGEN is designed to operate on externally supplied gene clusters, it is not restricted to K-means in principle. Future work can therefore examine whether more advanced clustering strategies, including graph-based, density-based, or stability-aware methods, further improve the biological quality of the resulting modules and, consequently, the interpretive performance of the framework.

Finally, BIOGEN is intended as an interpretive support layer rather than a discovery engine, and the current study does not include expert biological assessment or experimental validation of generated hypotheses. Future work should therefore extend the benchmark to expert-reviewed interpretations, broader biological resources, and experimental follow-up in order to better assess scientific utility in practice.

## 8 Conclusion

This research introduced BIOGEN as a multi-agent framework for evidence-grounded interpretation of RNA-seq transcriptional modules. Instead of framing post hoc biological interpretation as unconstrained natural-language generation, BIOGEN formalizes it as a retrieval-guided and verification-aware inference process grounded in external biomedical evidence. This design prioritizes source traceability, explicit evidential support, and reproducible interpretation of cluster-level biological signals.

The results show that BIOGEN is most appropriately viewed as an interpretive support layer that complements, rather than replaces, standard transcriptomic analysis workflows. Conventional enrichment analysis remains valuable for identifying canonical pathway associations, but it does not always provide sufficient cluster-level mechanistic context. BIOGEN addresses this gap by organizing heterogeneous biomedical evidence into structured explanations that make supporting sources, confidence levels, and evidential limitations more explicit.

Across five bacterial RNA-seq datasets evaluated under a shared pipeline configuration, BIOGEN consistently produced zero ungrounded outputs under the strict identifier-based criterion and preserved evidence-grounded interpretation under organism and condition shifts. In controlled comparison with representative open-source agentic AI frameworks, BIOGEN was the only framework to produce zero non-verifiable identifier outputs across all datasets, highlighting that retrieval access alone is not sufficient for scientifically useful interpretation unless the final output remains traceable to explicit supporting evidence.

Overall, these findings indicate that evidence-grounded agentic reasoning can serve a useful role in transcriptomic interpretation, particularly in settings where biological signals are diffuse, annotation coverage is incomplete, and manual contextualization is difficult to scale. The main contribution of BIOGEN therefore lies not in replacing existing statistical methods, but in providing a transparent and reproducible framework for connecting gene clusters to literature-supported biological context.

Future work should expand the framework through broader knowledge sources, more extensive expert-centered evaluation, improved organism-specific annotation support, and experimental follow-up of prioritized hypotheses. Three concrete extensions are already planned: (i) a manual citation-correctness audit on a stratified sample of cluster–citation pairs to assess the relevance and accuracy of cited identifiers beyond identifier-format presence; (ii) a sensitivity analysis over the per-cluster gene budget v∈{5,10,20} to quantify how representative-gene selection affects downstream interpretive metrics; and (iii) integration of structured signature resources such as MSigDB and organism-specific gene-set knowledge bases to reduce the dependence on PubMed indexing density and to broaden coverage for understudied genes.

## Data Availability

The original contributions presented in the study are included in the article/Supplementary Material, further inquiries can be directed to the corresponding author.
