# Supplementary material for: BIOGEN: evidence-grounded multi-agent reasoning framework for transcriptomic interpretation in antimicrobial resistance
Source: Front Bioinform. 2026 May 29;6:1846404. doi: 10.3389/fbinf.2026.1846404 (PMC13260171; doi:10.3389/fbinf.2026.1846404)
Supplement: Supplementary file 1 [file Supplementaryfile1.pdf]

Supplementary Material

BIOGEN: Evidence-Grounded Multi-Agent Reasoning Framework
for Transcriptomic Interpretation in Antimicrobial Resistance

This document provides supplementary material accompanying the paper *"BIOGEN: Evidence-Grounded Multi-Agent Reasoning Framework for Transcriptomic Interpretation in Antimicrobial Resistance"* (Hossain et al., *Frontiers in Bioinformatics*, 2026). All section, table, and figure numbers cited as "(main paper)" refer to the published manuscript.

Contents

A Framework Comparison Details **1**

B Model Configurations and Comparative Systems **2**

C Reproducibility and Environment **3**

D Runtime Summary **3**

E Per-Cluster and Per-Dataset BERTScore Decomposition **4**

F Hyperparameter and Configuration Summary **5**

G System and Agent Implementation Details **6**

G.1 Agentic Design Overview 6

G.2 Execution Pipeline 7

G.3 Agent Prompt Design 8

G.3.1 RetrieverAgent Prompt 8

G.3.2 InterpreterAgent Prompt 8

G.3.3 CriticAgent Prompt 8

G.4 Prompt Example and Model Output 8

H Source Code Availability **9**

A Framework Comparison Details

Table S1 reports the complete per-dataset, per-framework results underlying the compact summary presented in the main text. All systems were evaluated under the same controlled setup, using the same LLM backbone, retrieval sources, and cluster inputs, so that differences in output quality are attributable primarily to orchestration behavior rather than evidence access or model selection. The detailed results make the main tradeoff explicit: across all five datasets, BioGen consistently achieved the lowest identifier-based hallucination rate, whereas several generic agentic baselines obtained higher similarity-based scores such as BERTScore and SAS. This appendix table is therefore included to provide full transparency for the per-dataset metric values that support the discussion in Section 6.6 (main paper).

Table S1: Full controlled multi-dataset comparison of BioGen with four open-source agentic AI frameworks. All systems used the same LLM backbone, retrieval sources, and cluster inputs. N/A indicates that the corresponding metric was not computed in that setting.

Dataset	Framework	BERTScore ↑	SAS ↑	KEGG Sim ↑	Identifier-based hallucination ↓
PRJEB67574	BioGen	0.689	0.715	0.342	0.000
PRJEB67574	LangChain ReAct	0.727	0.808	0.407	0.600
PRJEB67574	CrewAI	0.726	0.761	0.443	0.500
PRJEB67574	AutoGen	0.733	0.841	0.437	0.300
PRJEB67574	Smolagents	0.736	0.822	0.422	0.600
GSE251671	BioGen	0.561	-0.016	N/A	0.000
GSE251671	LangChain ReAct	0.564	0.009	N/A	0.800
GSE251671	CrewAI	0.602	0.053	N/A	0.800
GSE251671	AutoGen	0.579	0.022	N/A	0.800
GSE251671	Smolagents	0.563	0.005	N/A	0.300
GSE144604	BioGen	0.715	0.504	N/A	0.000
GSE144604	LangChain ReAct	0.724	0.528	N/A	0.100
GSE144604	CrewAI	0.729	0.545	N/A	0.400
GSE144604	AutoGen	0.714	0.510	N/A	0.400
GSE144604	Smolagents	0.721	0.500	N/A	0.400
GSE224463	BioGen	0.550	-0.003	0.154	0.000
GSE224463	LangChain ReAct	0.629	0.095	N/A	1.000
GSE224463	CrewAI	0.630	0.050	N/A	0.800
GSE224463	AutoGen	0.609	0.020	N/A	0.900
GSE224463	Smolagents	0.636	0.075	N/A	1.000
GSE55197	BioGen	0.699	0.195	N/A	0.000
GSE55197	LangChain ReAct	0.724	0.507	N/A	0.600
GSE55197	CrewAI	0.727	0.504	N/A	0.300
GSE55197	AutoGen	0.728	0.573	N/A	0.400
GSE55197	Smolagents	0.722	0.525	N/A	0.400

B Model Configurations and Comparative Systems

To benchmark the individual and combined contributions of retrieval and critic mechanisms on the primary dataset, we evaluate four core system configurations.

- **LLM only (Mistral-7B).** This configuration uses the local Mistral-7B-Instruct-v0.2 model without retrieval or critic augmentation. All interpretations are generated solely from the model's parametric knowledge, providing a matched no-retrieval baseline.
- **LLM + Retrieval.** This configuration augments the same LLM with PubMed and UniProt retrieval before generation. The model receives retrieved literature and protein annotations as context, but no explicit verification or reliability scoring is applied.
- **SimpleRAG.** This configuration provides a strong single-agent retrieval-grounded baseline. It uses the same LLM backbone, the same representative genes per cluster, and the same PubMed and UniProt evidence sources as BioGen, but performs only a single interpretation step without critic-based verification or evidence-tier assignment.
- **BioGen (Full Framework).** The full system incorporates retrieval and all three critic modules (Evidence-Strict, Semantic, Adversarial) operating under data-adaptive evidence-tier thresholds derived from the empirical score distribution (Section 5.3, main paper). This configuration performs systematic evidence validation and reasoning-quality assessment, representing the complete BioGen pipeline.

All four configurations use the same local LLM backbone, the same clustering outputs, and the same per-cluster gene selection procedure, ensuring that differences in evaluation metrics

reflect the contribution of retrieval, single-agent synthesis, and critic-based verification rather than changes in backbone model or evidence access.

In addition to these system configurations, the final paper also reports a controlled comparison against four open-source agentic AI frameworks: LangChain ReAct, CrewAI, AutoGen, and Smolagents. In that multi-dataset comparison, all frameworks used the same shared LLM backbone, the same retrieval sources, and the same cluster inputs, so that agentic orchestration remained the primary experimental variable.

C Reproducibility and Environment

All experiments were executed locally on a workstation equipped with two NVIDIA TITAN RTX GPUs (24 GB VRAM each). GPU 1 was designated for all inference runs to leave GPU 0 available for other processes. The LLM backbone was loaded in 4-bit NF4 quantization using BitsAndBytes, requiring approximately 5 GB VRAM per session. The pipeline is reproducible at the script level: the main experimental runners accept CLI arguments such as `-data`, `-results`, and `-kegg`, enabling re-execution on different datasets without modifying source code. Key dependencies are listed in Table S2.

Table S2: Computational environment and software dependencies used in the reported experiments. All packages are version-locked in `requirements.txt`.

**Component**	**Version / Description**
Python	3.11
Transformers	4.41.2
BitsAndBytes	0.43.0 (4-bit NF4 quantization)
Sentence-Transformers	2.6.1
bert-score	0.3.x
gseapy	1.1.0
scipy	1.11.x (Fisher's exact test)
statsmodels	0.14.x (BH correction)
BioPython	1.83 (PubMed retrieval via Entrez)
LLM Backbone	Mistral-7B-Instruct-v0.2 (local, 4-bit NF4)
GPU	2× NVIDIA TITAN RTX, 24 GB VRAM

D Runtime Summary

Table S3 reports approximate runtime per cluster on the primary *Salmonella enterica* dataset for the four evaluated core system configurations. These values are intended to characterize relative computational overhead under the shared local environment described in Appendix C. As expected, retrieval introduces moderate additional cost relative to single-pass generation, while the full BioGen pipeline incurs further overhead from critic-based verification. In the current setup, the main runtime increase over retrieval-only generation arises from the critic stage.

These runtime values should be interpreted as approximate configuration-level estimates rather than exact wall-clock benchmarks for every dataset and framework. The multi-dataset framework comparison in the main paper is therefore analyzed primarily through output quality and grounding behavior, while runtime is included here as supporting reproducibility information.

Table S3: Approximate runtime summary on the primary *Salmonella enterica* dataset (10 clusters). All values are reported in seconds. "/C" denotes average time per cluster. The estimates are parameter-derived under the shared local hardware environment and are intended to reflect relative system overhead rather than exact wall-clock benchmarking.

**System**	**Ret./C**	**Gen./C**	**Crit./C**	**Total/C**	**Total×10**
LLM only	0.0	25.0	0.0	25.0	250
LLM + Retrieval	18.4	25.0	0.0	43.4	434
SimpleRAG	0.0	25.0	0.0	25.0	250
BioGen (Full Framework)	18.4	25.0	8.0	51.4	514

E Per-Cluster and Per-Dataset BERTScore Decomposition

Tables S4 and S5 report the per-cluster BERTScore Precision, Recall, F_1, and Semantic Alignment Score for the primary *Salmonella enterica* dataset. They also report the per-dataset BERTScore Precision, Recall, and F_1 decomposition across all five datasets. These tables were added in revision in response to reviewer requests to link the qualitative example in Figure 2 (main paper) to its underlying numerical metrics, and to separate BERTScore F_1 into precision and recall components so that dataset-level differences can be examined more directly.

The per-cluster Precision, Recall, F_1, and SAS values were recomputed using the same evaluation backbones employed elsewhere in the paper: `distilbert-base-uncased` for BERTScore and `all-MiniLM-L6-v2` for SAS. The per-cluster aggregate for the primary dataset, $F_1 \approx 0.726$, is slightly higher than the run-aggregated value reported in Table 2 of the main paper (0.689). This small difference reflects run-to-run variation between the canonical primary run and the outputs used for the per-cluster decomposition, while the relative ordering of clusters within the run remains unchanged.

Table S4: Per-cluster BERTScore (Precision, Recall, F_1) and Semantic Alignment Score for the primary *S. enterica* dataset (PRJEB67574). Cluster 0 corresponds to the example shown in Figure 2(a) (main paper). All values are computed against the same retrieval-pool reference text used in the run-aggregated metrics elsewhere in the paper.

**Cluster**	**BERT-P** ↑	**BERT-R** ↑	**BERT-F_1** ↑	**SAS** ↑
0	0.695	0.713	0.704	0.520
1	0.663	0.716	0.689	0.540
2	0.717	0.710	0.714	0.613
3	0.721	0.842	0.777	0.705
4	0.725	0.718	0.721	0.647
5	0.705	0.765	0.734	0.710
6	0.728	0.726	0.727	0.646
7	0.716	0.745	0.730	0.645
8	0.711	0.691	0.701	0.326
9	0.719	0.806	0.760	0.642
**Mean**	**0.710**	**0.743**	**0.726**	**0.599**

Two observations follow from Table S5. First, on three datasets (PRJEB67574, GSE144604, GSE55197), Precision and Recall are within 0.03 of each other, indicating roughly balanced overlap between the interpretation and the reference text. Second, on the two datasets with low

Table S5: Per-dataset BERTScore decomposition into Precision, Recall, and F_1 across the five evaluated datasets. The decomposition shows that on `GSE251671` and `GSE224463`, the comparatively low F_1 values arise primarily from low Precision (interpretations longer than the retrieval-pool reference) rather than from low Recall. The interpretations on these datasets cover most of the reference content (Recall ≥ 0.67) but also introduce additional context not present in the abstracts, which lowers Precision. We report this as a candidate explanation for the F_1 pattern; we do not claim that low literature support is the sole cause.

**Dataset**	n	**BERT-P** ↑	**BERT-R** ↑	**BERT-F$_1$** ↑
PRJEB67574 (*S. enterica*)	10	0.710	0.743	0.726
GSE144604 (*E. coli* MG1655)	10	0.704	0.727	0.716
GSE55197 (*P. aeruginosa* PA14)	4	0.726	0.746	0.736
GSE251671 (*P. aeruginosa* PA14)	10	0.470	0.702	0.562
GSE224463 (*E. coli* K-12)	10	0.467	0.672	0.551

F_1 (GSE251671 and GSE224463), Recall remains in the same 0.67–0.70 range as on the high-F_1 datasets, while Precision is much lower at ∼0.47. This precision drop, rather than a recall drop, is what drives the F_1 difference. The pattern is consistent with the interpretations on these datasets covering most of the retrieved-evidence content but also introducing biological context that is not present in the corresponding abstract pool. The reviewer's intuition that "weak literature support" is unlikely to be the explanation for *E. coli* K-12 (`GSE224463`) is consistent with this Recall pattern.

F Hyperparameter and Configuration Summary

Table S6 summarizes the key hyperparameters governing cluster construction, evidence retrieval, critic evaluation, and interpretation generation. The values reported below were chosen prior to metric computation and held fixed across all datasets and experiments to support reproducibility and to avoid post-hoc tuning. We provide explicit justification for the three parameters most likely to influence interpretive behavior: k (number of clusters), v (representative genes per cluster), and r (retrieved records per source per gene).

Number of clusters ($k = 10$). The cluster count was fixed at $k = 10$ based on the within-cluster inertia profile across $k \in [3, 15]$ on the primary *S. enterica* dataset, where the inertia curve flattens around $k \approx 9$–11. We treated this as a structural choice intended to give the framework a tractable number of cluster-level interpretations under the runtime constraints described in Appendix D. Because BioGen operates on externally supplied gene clusters, k is a property of the upstream clustering procedure rather than of the interpretive layer; downstream metrics therefore reflect the quality of cluster-level evidence grounding given this fixed partition. A formal sensitivity sweep over k, organism-specific elbow re-derivation, and stability-aware clustering alternatives are flagged as future work in Section 7 (main paper).

Representative genes per cluster ($v = 10$). The per-cluster gene budget was set to $v = 10$ rather than to the entire cluster for two reasons. First, retrieval is rate-limited at the API level (NCBI Entrez and UniProt REST both impose query-rate constraints), and a per-cluster budget bounds the retrieval cost from above. Second, the local Mistral-7B-Instruct-v0.2 backbone in 4-bit NF4 quantization has a finite effective context that must accommodate ten gene-level evidence blocks plus instructions; $v = 10$ leaves enough margin for the structured prompt and the multi-section output template. Within each cluster, the top-v genes are selected by within-cluster variance to prioritize locally informative signals rather than by global variance.

Retrieved records per source per gene ($r = 3$). The retrieval limit of $r = 3$ records

per source (PubMed and UniProt) per gene was chosen as a compromise between evidence breadth and downstream prompt-construction cost. A higher r would broaden context coverage but increases the risk that top-ranked PubMed hits contribute organism-level rather than gene-specific evidence; the Evidence Specificity Score analysis in Section 6.2 (main paper) shows that gene-specific signals are already sparse at $r = 3$, suggesting that larger r would not by itself increase specificity. We treat r as a fixed operational parameter rather than as a tuned hyperparameter.

Per-cluster gene selection uses within-cluster variance (top $v = 10$ genes per cluster) rather than global variance, ensuring each cluster is represented by its own most informative genes. Evidence-tier thresholds are derived automatically from the 33rd and 66th percentiles of the consensus score distribution and are not manually tuned. The KEGG organism code is passed as a CLI argument (`-kegg`) to support multi-organism evaluation without modifying source code.

We did not perform a separate $v \in \{5, 10, 20\}$ sensitivity sweep at the time of submission because the retrieval and generation cost scales roughly linearly with v across all five datasets, and a full sweep would require re-running the entire pipeline three times per dataset (15 full runs). We acknowledge this limitation explicitly and have flagged it as a planned follow-up analysis. The within-run robustness of evidence-tier assignments to nearby percentile thresholds (Section 6.2, main paper; Cohen's $\kappa = 1.000$ across p25/p75, p33/p66, p40/p60) provides indirect evidence that small perturbations to the configuration do not disrupt the qualitative behavior of the framework, but this does not substitute for a direct v sweep.

Table S6: Key hyperparameters used in the BioGen framework. Evidence-tier thresholds are data-adaptive (percentile-based) rather than fixed, and are re-derived independently for each dataset.

**Parameter**	**Value / Description**
Number of clusters (k)	10 (elbow method on inertia)
Top variable genes per cluster (v)	10 (within-cluster variance)
Clustering target	Genes (transposed expression matrix)
Max evidence per gene (r)	3 (PubMed) + 3 (UniProt)
Evidence tier method	Percentile tertile split (p33 / p66)
Critic thresholds	$\theta_{\text{evidence}} = 0.7$, $\theta_{\text{semantic}} = 0.6$, $\theta_{\text{adversarial}} = 0.5$ (per-critic operating points; see Section 4.3, main paper)
LLM temperature	0.2
Retrieval batch size	8
Embedding model	Sentence-BERT (`all-MiniLM-L6-v2`)
LLM quantization	4-bit NF4 (BitsAndBytes)
Default KEGG organism code	`stm` (*S. enterica* LT2)

G System and Agent Implementation Details

G.1 Agentic Design Overview

BioGen is implemented as a modular agentic pipeline in which each agent performs a well-defined, verifiable role. The architecture comprises four primary components: the **ClusterAgent**, which performs gene-level unsupervised clustering on the transposed expression matrix; the **RetrieverAgent**, which interfaces with PubMed and UniProt via the NCBI Entrez API and the UniProt REST API; the **InterpreterAgent**, which synthesizes retrieved evidence into

structured biological reasoning using the local Mistral-7B-Instruct-v0.2 backbone; and the **CriticAgent ensemble**, which evaluates interpretive reliability through three complementary validation perspectives.

All agents communicate through structured JSON messages under a central orchestration controller that coordinates task scheduling, evidence caching, and output harmonization. Intermediate retrievals, interpretations, and critic scores are cached to ensure traceability and avoid redundant API calls. This design enables each agent to be re-executed independently without affecting upstream or downstream dependencies.

Table S7: Functional overview of the BioGen agent ecosystem.

**Agent**	**Functionality**
ClusterAgent	Performs gene-level K-means clustering (k=10) on the transposed expression matrix and identifies per-cluster top-variable genes (v=10, within-cluster variance) for downstream interpretation.
RetrieverAgent	Queries PubMed (NCBI Entrez) and UniProt REST API using locus-tag-based gene identifiers, returning structured evidence records with specificity labels (specific / generic).
InterpreterAgent	Generates structured cluster-level biological interpretations using a local Mistral-7B-Instruct-v0.2 backbone (4-bit NF4), grounding outputs in retrieved evidence and reporting evidence-linked explanations with explicit limitations.
CriticAgents	Three complementary critics – Evidence-Strict (identifier-level factual validation), Semantic (Sentence-BERT embedding consistency), and Adversarial (LLM-based counterfactual challenge) – whose scores are aggregated by majority voting into a consensus reliability indicator.

G.2 Execution Pipeline

The BioGen pipeline follows a two-pass deterministic sequence designed to ensure that evidence-tier labels are globally consistent within each run.

1. **Pass 1 – Clustering, retrieval, interpretation, and scoring.** The ClusterAgent partitions the transposed expression matrix into gene-level transcriptional modules. For each cluster, the RetrieverAgent collects PubMed and UniProt evidence, the InterpreterAgent generates a structured hypothesis, and the CriticAgent ensemble produces a consensus score. All consensus scores are collected before any tier assignment.

2. **Pass 2 – Data-adaptive tier assignment.** After all clusters are processed, evidence-tier boundaries are derived from the full score distribution using the 33rd and 66th percentiles. Each interpretation is labeled with its evidence tier, confidence score, and supporting references, then saved to the result files associated with that run.

This two-pass design ensures that tier boundaries are consistent across the entire run and that threshold derivation is data-adaptive rather than predetermined by a fixed constant.

G.3 Agent Prompt Design

Each agent employs a structured prompt template standardized across experimental runs to maintain reproducibility and allow consistent benchmarking across datasets.

G.3.1 RetrieverAgent Prompt

RetrieverAgent Prompt Template

Task: Retrieve biomedical literature for a given gene from a bacterial RNA-seq cluster.
Instructions: 1. Query PubMed for exact gene/protein mentions (max 3 results per gene).
2. Retrieve UniProt entries for each gene (max 3 per gene).
3. Broaden to "<gene> AND <organism>" if no specific hits are found.
Output JSON: {"gene": "...", "source": "PubMed/UniProt", "title": "...", "pmid": "...", "category": "specific/generic"}

G.3.2 InterpreterAgent Prompt

InterpreterAgent Prompt Template

Task: Generate a structured cluster-level biological interpretation grounded in retrieved evidence.
Instructions: 1. Identify functional themes (e.g., virulence, efflux, stress response).
2. Summarize putative pathways and regulatory mechanisms supported by evidence.
3. Cite PubMed IDs and UniProt accessions inline for every claim whenever available.
4. Report limitations explicitly where evidence is incomplete or generic.
Output sections: Functional themes / Putative pathways / Regulation / Key genes and evidence / References / Confidence / Limitations

G.3.3 CriticAgent Prompt

CriticAgent (Adversarial) Prompt Template

Task: Critically evaluate the InterpreterAgent output.
Checks: 1. Are all claims in the interpretation supported by cited PubMed or UniProt evidence?
2. Are references organism-appropriate for the dataset under analysis?
3. Is the reported confidence score consistent with citation density and quality?
Output JSON: {"score": 0.0–1.0, "reliable": true/false}

G.4 Prompt Example and Model Output

Example Input/Output for Cluster 1 (*S. enterica*)

Input genes: gene-PSLT017 (pefC), gene-PSLT039 (spvB), gene-STM3755 (rhuM)
Retrieved evidence: PubMed 40373749 – "A Salmonella subset exploits erythrophagocytosis to subvert SLC11A1-imposed iron deprivation." UniProt P37868 – "Outer membrane usher protein PefC." UniProt H9L477 – "Mono(ADP-ribosyl)transferase SpvB."
BioGen Output: {"themes": "Plasmid-encoded virulence and iron acquisition", "pathways": "Fimbrial assembly (pefC), ADP-ribosylation (spvB), erythrophagocytosis subversion", "references": ["40373749", "P37868", "H9L477"], "confidence": 0.45, "evidence_tier": "High confidence", "limitations": "PubMed hits are organism-level generic; locus names absent from retrieved titles"}

H Source Code Availability

The code can be shared for scientific and academic research purposes upon reasonable request to the authors.
